# Neonicotinoid Insecticides Alter the Gene Expression Profile of Neuron-Enriched Cultures from Neonatal Rat Cerebellum

**DOI:** 10.3390/ijerph13100987

**Published:** 2016-10-04

**Authors:** Junko Kimura-Kuroda, Yasumasa Nishito, Hiroko Yanagisawa, Yoichiro Kuroda, Yukari Komuta, Hitoshi Kawano, Masaharu Hayashi

**Affiliations:** 1 Department of Brain Development and Neural Regeneration, Tokyo Metropolitan Institute of Medical Science, Setagaya-ku, Tokyo 156-8506, Japan; hayashi-ms@igakuken.or.jp; 2 Center for Basic Technology Research, Tokyo Metropolitan Institute of Medical Science, Setagaya-ku, Tokyo 156-8506, Japan; nishito-ys@igakuken.or.jp; 3 Advanced Clinical Research Center, Institute of Neurological Disorders, Kawasaki, Kanagawa 215-0026, Japan; hiro-yanagisawa@jikei.ac.jp; 4 Environmental Neuroscience Information Center, Musashino, Tokyo 180-0014, Japan; yo-kuroda@nifty.com; 5 Division of Neurology, Department of Internal Medicine, National Defense Medical College, Tokorozawa, Saitama 359-8513, Japan; con376@ndmc.ac.jp; 6 Department of Health & Dietetics, Teikyo Heisei University, Toshima-ku, Tokyo 170-8445, Japan; kawano.hitoshi3@gmail.com

**Keywords:** pesticide, neonicotinoid, imidacloprid, acetamiprid, developmental neurotoxicity, microarray, transcriptome, cerebellar culture, brain development

## Abstract

Neonicotinoids are considered safe because of their low affinities to mammalian nicotinic acetylcholine receptors (nAChRs) relative to insect nAChRs. However, because of importance of nAChRs in mammalian brain development, there remains a need to establish the safety of chronic neonicotinoid exposures with regards to children’s health. Here we examined the effects of long-term (14 days) and low dose (1 μM) exposure of neuron-enriched cultures from neonatal rat cerebellum to nicotine and two neonicotinoids: acetamiprid and imidacloprid. Immunocytochemistry revealed no differences in the number or morphology of immature neurons or glial cells in any group versus untreated control cultures. However, a slight disturbance in Purkinje cell dendritic arborization was observed in the exposed cultures. Next we performed transcriptome analysis on total RNAs using microarrays, and identified significant differential expression (*p* < 0.05, *q* < 0.05, ≥1.5 fold) between control cultures versus nicotine-, acetamiprid-, or imidacloprid-exposed cultures in 34, 48, and 67 genes, respectively. Common to all exposed groups were nine genes essential for neurodevelopment, suggesting that chronic neonicotinoid exposure alters the transcriptome of the developing mammalian brain in a similar way to nicotine exposure. Our results highlight the need for further careful investigations into the effects of neonicotinoids in the developing mammalian brain.

## 1. Introduction

Neonicotinoids are used worldwide as pesticides, but their toxicity also affects beneficial insects, such as honey bees, and other invertebrate species [1,2]. Neonicotinoids are agonists of nicotinic acetylcholine receptors (nAChRs) and their effectiveness has been attributed to their high affinity for insect nAChRs. Furthermore, their low affinity for several subsets of mammalian nAChRs in binding assays suggests that neonicotinoids are relatively safe for mammals including humans [3,4]. However, there have been reports of severe or fatal cases of poisoning by the neonicotinoids imidacloprid (IMI), acetamiprid (ACE), and thiacloprid [5,6,7]. Marfo et al. reported the occurrence of subacute neonicotinoid intoxication through daily consumption of contaminated food in Japan [8]. They detected high levels of neonicotinoid metabolites in the urine of patients with symptoms typical of neo-nicotinoid intoxication, including headache, general fatigue, palpitation/chest pain, abdominal pain, muscle pain/weakness/spasm, cough, finger tremor, and short-term memory loss. Furthermore, an in vitro study reported that IMI and clothianidin agonize human α4β2 nAChR and disrupt receptor responses against the endogenous ligand acetylcholine (ACh) [9]. Moreover, some metabolites of the neonicotinoids have a high affinity for mammalian nAChRs, similar to that of nicotine (NIC) [3]. Together, these findings suggest that neonicotinoids might be a risk to human health, but there remains a paucity of data on this subject [10].

In rodent experiments, exposure to IMI in utero was shown to cause impairments in sensorimotor performance and overexpression of glial fibrillary acidic protein (GFAP) in the motor cortex and hippocampus of neonatal rats [11]. Furthermore, the neonicotinoids thiamethoxam and clothianidin induced dopamine release in the rat striatum via nAChRs [12] and thiamethoxam altered behavioral and biochemical processes related to the cholinergic systems in rat [13]. Gestational administration of clothianidin induces behavioral disorders in F1 mice [14] and exposure to clothianidin and other stress causes behavioral and reproductive abnormalities in male mice [15]. Exposure to IMI alters learning performance and related gene expression in infant and adult rats [16]. Recently, Sano et al. reported that exposure to low dose ACE in utero and through lactation induces abnormalities in socio-sexual and anxiety-related behaviors in male mice [17]. Additionally, an in vivo mouse study revealed that four types of neonicotinoids (ACE, IMI, thiacloprid, and nitenpyram) readily pass through the blood–brain barrier (BBB) and are detectable in the brain at 60%–70% of the serum concentration within 15 min of intraperitoneal administration [18].

We previously reported that excitatory Ca^2+^-influx was evoked by NIC, ACE, and IMI at concentrations greater than 1 μM in small neurons in neonatal rat cerebellar cultures expressing α3, α4, and α7 nAChR subunit mRNA [19]. The proportion of excited neurons, and peak excitatory Ca^2+^-influx induced by ACE and IMI were lower than those induced by NIC. However, ACE and IMI had greater effects on mammalian neurons than would be predicted based on the results of binding assay studies. Because three nAChR antagonists significantly inhibited ACE- and IMI-induced neuronal Ca^2+^-influx, it is likely that ACE and IMI have direct agonist activity at nAChRs in cerebellar neurons.

During mammalian brain development, expression of several nAChR subtypes is higher during the fetal stage than at the mature stage, and are important for synapse formations. In the human fetal cerebellum, α4, α7, β2, and β4 nAChRs are highly expressed [20]. Recently, several lines of evidence indicated that endogenous cholinergic signaling via nAChRs is important in determining the morphological and functional maturation of neural circuit formation [21]. Glutamatergic synapse formation is promoted by α7-containing nAChRs and affected by NIC exposure in hippocampal and cortical neurons [22]. Retinal β2 nAChRs are necessary for visual circuit formation [23] and prenatal NIC exposure alters the visual cortex system in baboons [24]. Even at a dose lower than that necessary to activate the receptor, NIC causes desensitization of nAChRs [25], which results in a disturbance of normal synapse formations at the developmental stage [26].

Although neonicotinoids have a lower affinity for mammalian nAChRs than for insect nAChRs, they have similar chemical characteristics to NIC, which is known to disrupt normal brain development. Therefore, their effects on human health especially in the developing brain must be carefully examined. In 2013, the European Food Safety Authority proposed that ACE and IMI had the potential to be developmentally neurotoxic [27]. However, the impact of neonicotinoids on mammalian nAChRs and brain development has not been sufficiently studied.

In the present study, we examined whether chronic neonicotinoid exposure affects the gene expression profile in the developing mammalian brain using microarrays of neuron-enriched cultures from neonatal rat cerebellum. Transcriptome profile analysis is a useful tool to demonstrate possible effects of neonicotinoids on multiple nAChR functions. Rodent neonatal cerebellar cultures are commonly used for studying developing neurons [28]. Maturation of the rat cerebellum takes about 30 days (from the 12-day embryo to postnatal day (P) 19) [29]. The cerebellum is used to model important neurodevelopmental processes in vitro, such as neuronal cell differentiation, neurite outgrowth, and synapse formation. Therefore, we used neuron-enriched cultures derived from cerebella from 1-day-old rats, with or without exposure to NIC, ACE, or IMI, and identified significant differential expression of several genes important for brain development.

## 2. Materials and Methods

### 2.1. Chemicals

(−)-Nicotine (NIC, purity >99%; Sigma-Aldrich, St Louis, MO, USA), imidacloprid (IMI, 1-(6-chloro-3-pyridylmethyl)-N-nitroimidazolidin-2-ylideneamine, purity >98%; Kanto Chemicals Inc., Tokyo, Japan), and acetamiprid (ACE, (*E*)-N1-[(6-chloro-3-pyridyl)methyl]-N2-cyano-N1-methyl-acetamidine, purity >98%; Wako Chemicals Inc., Osaka, Japan),) were dissolved in dimethyl sulfoxide (DMSO, Sigma-Aldrich) to 100 mM stock solutions, and stored at −30 °C immediately prior to use.

### 2.2. Animals and Ethics Statement

Gestational Sprague-Dawley rats were purchased from Clea Japan, Inc. (Tokyo, Japan). All experimental procedures were performed in accordance with and approved by the Animal Use and Care Committee of the Tokyo Metropolitan Institute of Medical Science (permit no. 15003), and all animals were cared for and treated humanely in accordance with our institutional animal experimentation guidelines.

### 2.3. Neuron-Enriched Cultures of Neonatal Rat Cerebellum

The details of the culture methods have been described previously [19]. In brief, cerebella of P1 neonatal rats were digested with papain, and dissociated cells were suspended in a synthetic culture medium containing 1% fetal bovine serum. The cells were plated at a density of 1.2 × 10^6^ cells/0.8 mL in a chamber slide well (4 cm^2^/well, Permanox, Nunc Lab-Tek, Sigma-Aldrich) that was precoated with 0.1 mg/mL poly-L-lysine (Sigma-Aldrich) and 10 μg/mL laminin (BD Biosciences, Franklin Lakes, NJ, USA). Four wells (4.8 × 10^6^ cells) per group (control, NIC, ACE, and IMI) were used for each experiment. The remaining cerebellar cells were plated in chamber slide wells (0.7 cm^2^/well) at the same density as the 4 cm^2^ wells, cultured in the same manner, and then used for immunocytochemistry.

After 2 days, NIC, ACE, or IMI (final concentration 1 μM), or DMSO (control; final concentration 0.001%) was added to the cell cultures at a final concentration of 1 μM with 0.8 mL of 1% fetal bovine serum. Every 2 days for the 16 days in vitro (DIV), half of the culture medium was replaced with fresh medium containing 1μM of NIC, ACE or IMI, or 0.001% DMSO. After 8 DIV, serum-free synthetic medium was used to prevent the growth of astrocytes. The serum-free synthetic medium consisted of Dulbecco’s modified Eagle medium/F12 (Gibco, Thermo Fisher Scientific, Waltham, MA, USA) with 10 μg/mL bovine insulin (Sigma-Aldrich), 100 μg/mL transferrin (Sigma-Aldrich), 30 nM sodium selenite, 5 nM thyroxine, 100 μM putrescine (Sigma-Aldrich), and penicillin-streptomycin (100 units/mL and 100 µg/mL, respectively; Gibco).

We performed six independent culture experiments. For each experiment, cultures for all four groups were prepared from the same lot of cerebellar cells (from 12–15 neonates of one litter).

### 2.4. RNA Preparation

Total RNA was isolated from 16 DIV cerebellar cells using TRIzol reagent (Invitrogen, Thermo Fisher Scientific) and the Direct-zol RNA Miniprep Kit (Zymo Research, Irvine, CA, USA) following the manufacturers’ recommendations. Quality of the total RNA was strictly controlled: by several parameters. The RNA extracts were analyzed by a Nanodrop 2000 spectrophotometer (Thermo Fisher Scientific, Waltham, MA, USA) was used to confirm RNA quality with an A260/A280 ratio close to 2 and an A260/A230 ratio greater than 2. In total, 24 samples were tested, comprising six samples for each of the four treatments: control, NIC, ACE, and IMI.

### 2.5. Microarray

RNA quality was further assessed using a 2100 Bioanalyzer (Agilent Technologies, Valencia, CA, USA). cDNA and cRNA were synthesized with a Low Input Quick Amp Labeling Kit (Agilent Technologies) according to the manufacturer’s instructions. The labeled cRNA samples were then hybridized to the Whole Rat Gene Expression ver.3 4x44k Microarray (G4847B; Agilent Technologies). The array slides were washed and scanned using a SureScan Microarray Scanner (Agilent Technologies). Microarray data were analyzed using Feature Extraction software v11.5.1.1 (Agilent Technologies).

### 2.6. Data Normalization and Quality Control

Data were analyzed using GeneSpring GX 13.1 software (Agilent Technologies). Raw signals were log transformed and normalized using the quantile normalization method. For each probe, the median of the log summarized values from all the samples was calculated and subtracted from each of the samples to get a transformed baseline. Quality control for each array was checked according to GeneSpring instructions. The final data set contained an expression signal above the 20th percentile in more than half the samples within range, so as not to exclude a particularly large change caused by some treatment. These microarray data have been submitted to the Gene Expression Omnibus and are accessible through accession number GSE 80656.

### 2.7. Differential Expression Analysis and Statistics

To determine the specific effects of NIC, ACE, and IMI, each treatment versus control was analyzed and the results were compared. Not all of the normalized array data followed a normal distribution, so we used the nonparametric paired Mann-Whitney U test; this reduced the variation within each culture lot (each from a single litter). Storey’s bootstrapping estimation of the false discovery rate (FDR; *q*) was used to correct for multiple comparisons [30]. Differences were considered statistically significant for *p* and *q* values <0.05, and genes with a 1.5 (log_2_ 0.585)-fold change (FC) were selected for analysis. For several genes, the arrays contained multiple probes on the chip. In these cases, we limited each gene to corroborate direction and magnitude of change. We also excluded the compromised data and the probes without gene symbols or descriptions.

### 2.8. Quantitative Real-Time PCR (qRT-PCR)

For both qRT-PCR and microarray analyses, the same RNA samples were used from six independent series of culture experiments. The cDNA was generated using ReverTra Ace qPCR RT Master Mix (Toyobo, Osaka, Japan). Ready-made primers of Cacna1h, Cacng1, Dcdc2, F2rl2, Gpr83, Htr2c, Mb, Mcmdc2, Myog, Mypn, Pcdhgb7, Tada2b, Tnni2, Txk, Bche, Atp5f1, and Unc45b were purchased from Takara (Shiga, Japan). Primers for B3gnt9, Cadm3, Lyn, Magel2, and Rbfox2 were designed using Primer3Plus [31] and NCBI primer-BLAST [32] and purchased from BEX (Tokyo, Japan) (supplemental Table S1). Primers for the housekeeping genes Actb and Gapdh were described previously [33,34] and purchased from BEX. The qRT-PCR reactions were prepared using THUNDERBIRD SYBR qPCR Mix (Toyobo), and performed using the Bio-Rad CXF96 Real-Time PCR Detection System (Bio-Rad, Hercules, CA, USA). The following conditions were used: 95 °C for 1 min, 39 cycles of 95 °C for 10 s and 60 °C for 40 s. The Pearson correction coefficient between the qRT-PCR and microarray was analyzed using GraphPad Prism 6 (GraphPad Software, San Diego, CA, USA).

To determine α3, α4, and α7 nAChR mRNA expression in the cultures, we performed qRT-PCR analysis of the genes encoding these subunits (Chrna3, Chrna4, and Chrna7, respectively; primers purchased from Takara) using the same RNA samples.

### 2.9. Gene Ontology and Bioinformatics Analysis

To identify the molecular functions and diseases associated with the differentially expressed (DE) genes after NIC, ACE, or IMI exposure, we used the Protein Analysis Through Evolutionary Relationships (PANTHER) Classification System [35] and MetaCore analysis software (Thomson Reuters, New York, NY, USA). Overviews of DE gene ontology (GO) were examined using PANTHER. With regard to MetaCore, because ontologies typically contain many terms, some of them may turn out to be significant for a particular list and given p-value. The enrichment p values vary dependent on the size of the gene list and the selection of the background. The enrichment analysis FDR was controlled using the Benjamini and Hochberg method. Each DE gene list was analyzed by MetaCore enrichment analysis including GO processes (gene functions) and related diseases to identify genes involved in neurodevelopment. Next, all three gene lists were analyzed by comparison experiment tools, which can be used for comparing experimental data by analyzing their intersections.

### 2.10. Immunocytochemistry

At 16 DIV, cultured cerebellar cells were fixed with 4% paraformaldehyde in 0.1 M phosphate buffer for 20 min at room temperature. The following primary antibodies were used to detect specific antigens: mouse monoclonal anti-Tuj1 (Covance, Princeton, NJ, USA) as a marker for immature neurons, mouse monoclonal anti-calbindin D28k (Sigma-Aldrich) as a Purkinje cell marker, rabbit anti-GFAP (Dako, Glostrup, Denmark) as an astrocyte marker, mouse anti-oligodendrocyte marker O4 (Chemicon, Merck Millipore, Billerica, MA, USA), and mouse anti-CD11b OX42 IgM (Serotec, Bio-Rad), as a microglial marker. For double-labeling, the cells were incubated with primary antibodies against TuJ1, O4, and OX42 or GFAP, then Alexa 488-labeled anti-mouse IgG or IgM (Molecular Probes, Thermo Fisher Scientific), and Alexa 594 labeled anti-rabbit IgG secondary antibodies (Molecular Probes). The nucleus was stained with Hoechst 33342 (Molecular Probes). The cells were observed by confocal laser microscopy (Zeiss, LSM780, Oberkochen, Germany). Percentages of neural cells were calculated from the number of immunostained cells per Hoechst-positive-nucleus (*n* = approximately 1000) across three to four experiments, using MetaMorph imaging software (Molecular Device, Sunnyvale, CA, USA). To measure the dendritic arborization of Purkinje cells, the calbindin D28k-immunoreactive area was quantified from 10 cells per sample, using MetaMorph, in three independent experiments, as described previously [36] (see Figure 2 for representative results).

## 3. Results

### 3.1. Morphological Characterics of 16 DIV Cerebellar Neuron-Enriched Cultures

**Figure 1:**
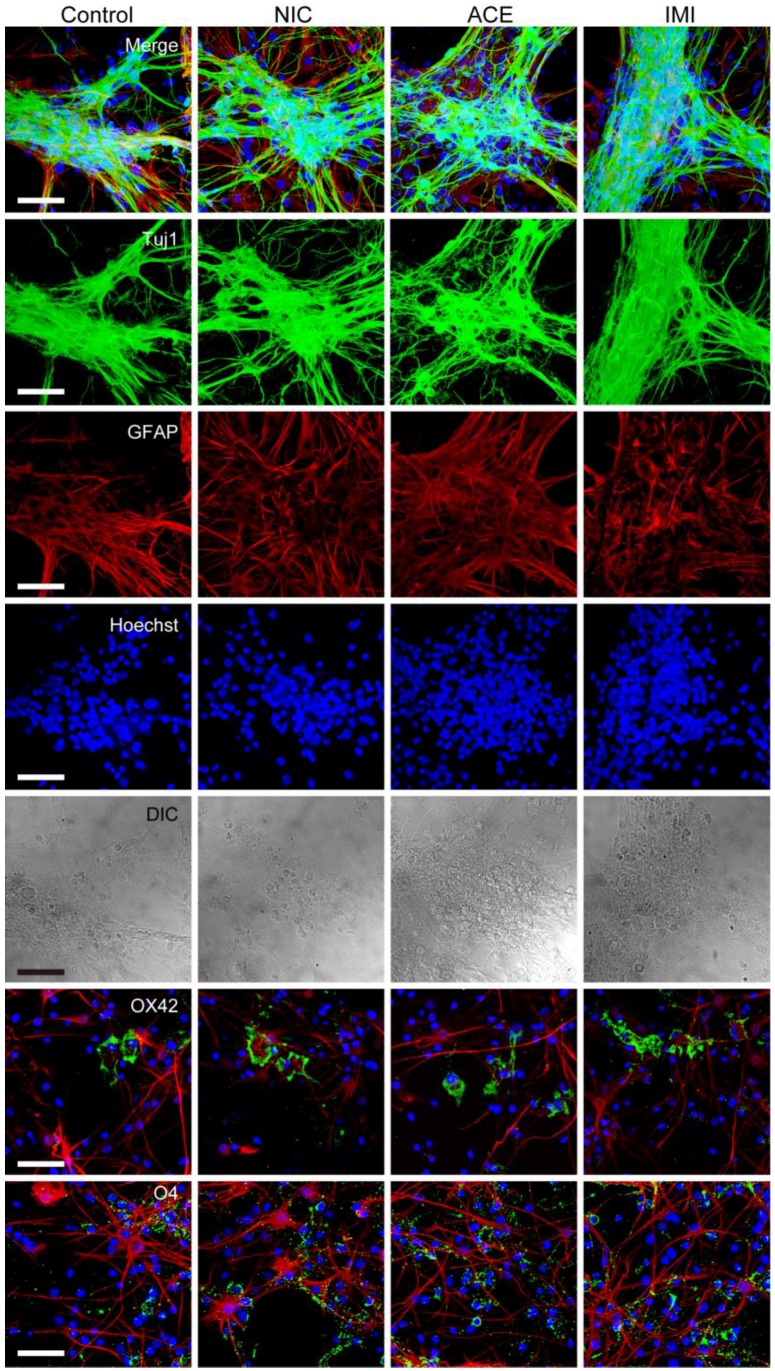
Morphology of cells in cerebellar cultures exposed to nicotine (NIC), acetamiprid (ACE), and imidacloprid (IMI) for 14 days (16DIV). Horizontal rows from top to bottom show merged images (Tuj1, glial fibrillary acidic protein (GFAP), and Hoechst); Tuj1-positive neurons; GFAP-positive astrocytes; Hoechst-positive nuclei; differential interference contrast (DIC) images; OX42-positive microglia (green); and O4-positive oligodendrocytes (green). From the first to the fifth row, the same sample is shown. In the OX42 and O4 rows, red represents GFAP and blue is Hoechst stain. The photos of microglia and oligodendrocytes were obtained from areas of sparse cell density, because their morphology was easy to identify. Bars = 50 μm. No notable morphological differences were observed between the groups.

**Figure 2:**
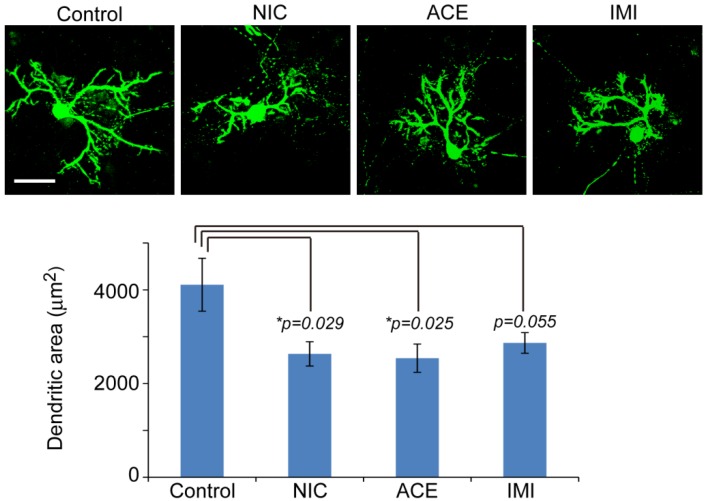
Dendritic arborization of Purkinje cells in cerebellar cultures exposed to nicotine (NIC), acetamiprid (ACE), and imidacloprid (IMI) for 14 days (16DIV). A slight disturbance in the dendritic arborization exposed to NIC, ACE, and IMI was observed. Upper panel shows a representative cell from each group stained for anti-calbindin D28k. Bar = 50 μm. Graph shows MetaMorph quantification of calbindin D28k-reactive dendritic area (without cell soma) (*n* = 10 cells per group). Error bar represent standard errors. *T*-tests were conducted for each treatment versus control. * *p* < 0.05.

To determine the effects of NIC, ACE, or IMI exposure on survival rate and morphological features of cells in the cerebellar cultures, we immunostained cerebellar cells for specific antibodies (Figure 1). We observed no notable morphological differences in Tuj1-positive neurons (large Purkinje cells, small granule cells, and other interneurons such as basket cells, Golgi cells, stellate cells), and glial cells (astrocytes, oligodendrocytes, and microglia) between the four groups in any of the six culture lots. However, staining for calbindin D28k to show the detailed morphology of Purkinje cells revealed a slight disturbance in the dendritic arborization of cells exposed to NIC, ACE, and IMI, and the reduction of dendritic area was 38%~40% in the treatment groups compared to a control (Figure 2). In each of the four groups in each experiment, the majority (approximately 61%) of cells were Tuj1-positive neurons. Other cells included approximately 28% GFAP-positive astrocytes, 5% O4-positive oligodendrocytes, and 3% OX42-positive microglia (for details see supplemental Figure S1). Other neural cells, which were not identified by staining, were likely to be NG2 glial cells or neural stem cells [37,38]. Hoechst staining revealed approximately 1% abnormal nuclei (indicating cell death).

### 3.2. Differential Gene Expression after Exposure to NIC, ACE and IMI

For control versus NIC (CvN), 4550 of 23,011 filtered probes were identified as significant (*p* < 0.05, *q* < 0.05), and were used for differential expression analysis (fold change (FC) ≥ 1.5). The 92 probes that passed were checked for multiple probes, gene symbol, descriptions, and of these 34 DE genes were selected (Table 1 and Table 2).

For control versus ACE (CvA), 4557 of 23,012 filtered probes were significant (*p* < 0.05, *q* < 0.05), of which 106 DE probes (FC ≥ 1.5) were checked as described above, and 48 DE genes were selected (Table 1 and Table 3).

For control versus IMI (CvI), 4862 of 22,852 filtered probes were significant (*p* < 0.05, *q* < 0.05), of which 162 DE probes (FC ≥ 1.5) were checked, and 67 DE genes were selected (Table 1 and Table 4).

**Figure 3:**
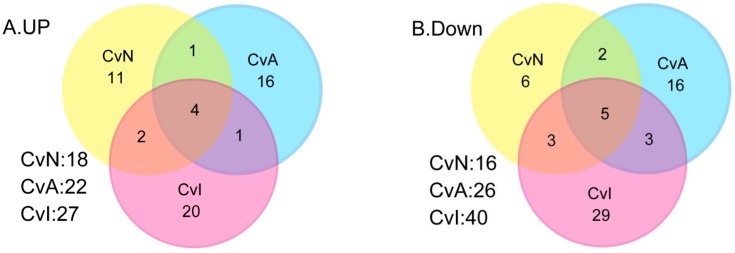
Overviews of gene expression changes in cerebellar cultures exposed to nicotine (NIC), acetamiprid (ACE), and imidacloprid (IMI) for 14days (16DIV). In altered transcriptomes of control versus NIC (CvN), control versus ACE (CvA), and control versus IMI (CvI), 34, 48, and 67 genes, respectively were filtered at cutoff threshold values of *p* < 0.05, *q* < 0.05, and fold change (FC) ≥ 1.5. Venn diagrams show numbers of genes upregulated (**A**) and downregulated (**B**) after the three treatments versus control.

Next, we constructed Venn diagrams to visually assess the overlap and separation between the three DE gene lists, CvN (34 genes), CvA (48 genes), and CvI (67 genes). As shown in Figure 3 and Table 1, nine genes (four upregulated and five downregulated versus control) were common to three gene lists. Common to at least two lists were three genes between CvN and CvA, five genes between CvN and CvI, and four genes between CvA and CvI.

**Figure 4:**
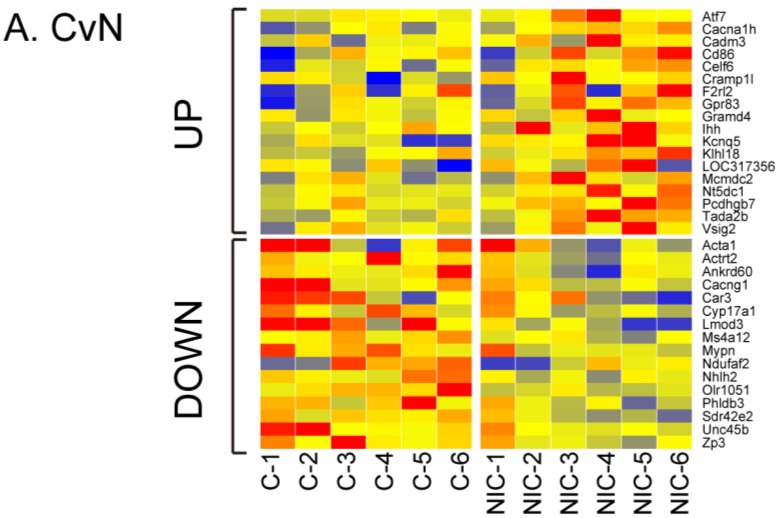

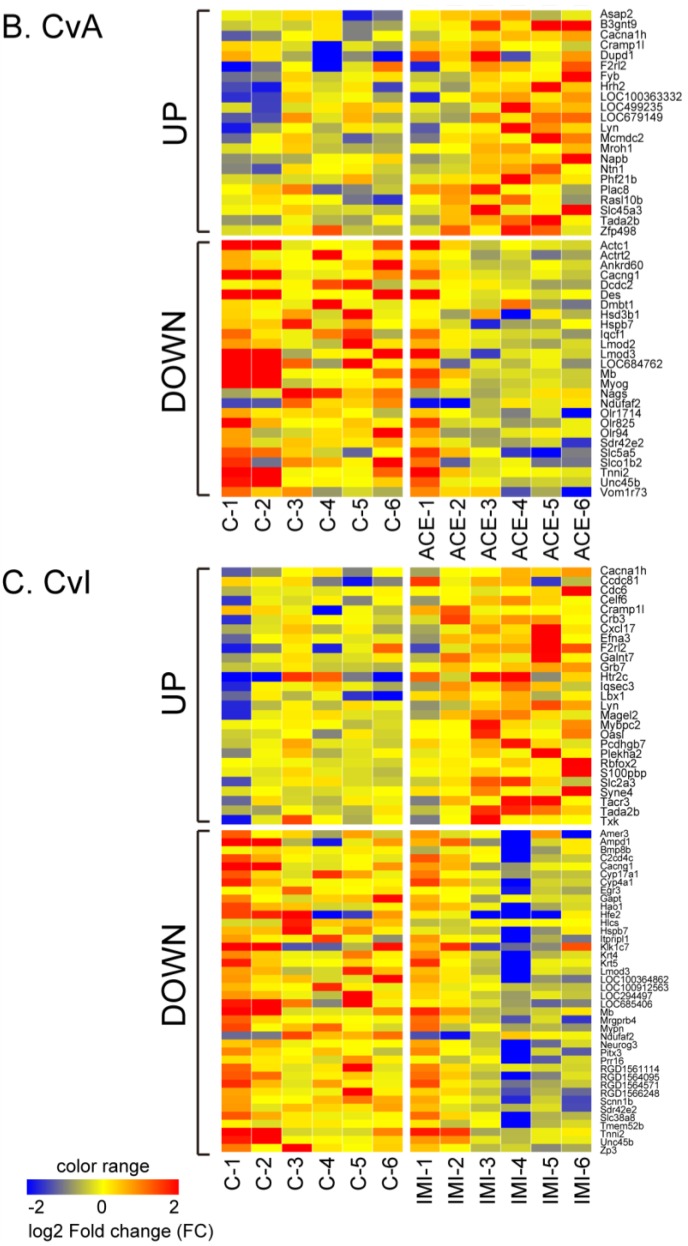
Heat maps of differentially expressed for control versus NIC (**A**), ACE (**B**), or IMI (**C**) (CvN, CvA, and CvI, respectively) from each of the six experiments (x-axis). Gene probes are listed alphabetically on the y-axis. Color change represents magnitude of log_2_ fold change (−2 (blue) to 2 (red)).

Other DE genes unique to CvN, CvA, or CvI are shown in Table 2, Table 3 and Table 4. The differential expression levels of the three groups are presented as heat maps and standard deviations that display expression differences between each of the treatments (NIC, ACE, and IMI) and control (Figure 4, Table S2).

### 3.3. Quantitative qRT-PCR Validation

**Figure 5:**
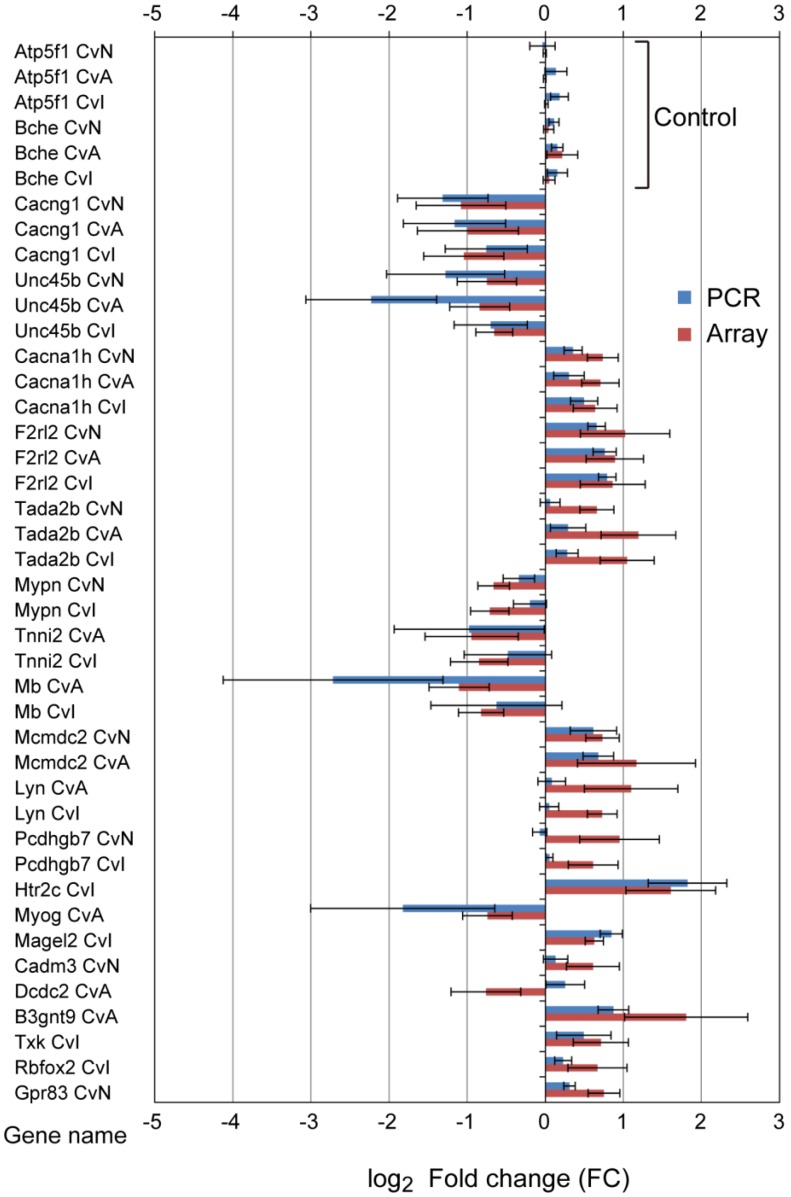
Confirmation of the microarray data using quantitative real-time PCR (qRT-PCR). Twenty DE genes and two control genes were selected at random for validation. Red bars, microarray data; blue bars, qRT-PCR data. The qRT-PCR data were normalized against the reference gene Actb; similar results were obtained using Gapdh as reference (not shown). The similarity of the expression patterns (up- and downregulation) between the microarray and qRT-PCR analyses confirmed the results of the microarray. Error bars represent standard errors from six experiments. Control genes’ descriptions are follows; Atp5f1, ATP synthase, H^+^ transporting, mitochondrial Fo complex, subunit B1; Bche, butyrylcholinesterase.

qRT-PCR was used to validate microarray data for 20 randomly selected DE genes and two control genes. Almost all genes showed consistent trends between qRT-PCR and microarray results (Figure 5), with the Pearson correction coefficient of 0.80 between the qRT-PCR and microarray data. In Tada2b, log_2_ FC of PCR was noticeably lower than those of microarray, especially CvN. Of the 22 genes selected, changes in only three (Lyn, Pcdhgb7, and Dcdc2) were not confirmed by qRT-PCR. This may be because of differences in primer positions, resulting in transcript variants or degradation of mRNA.

### 3.4. nAChRs Expressions

**Figure 6:**
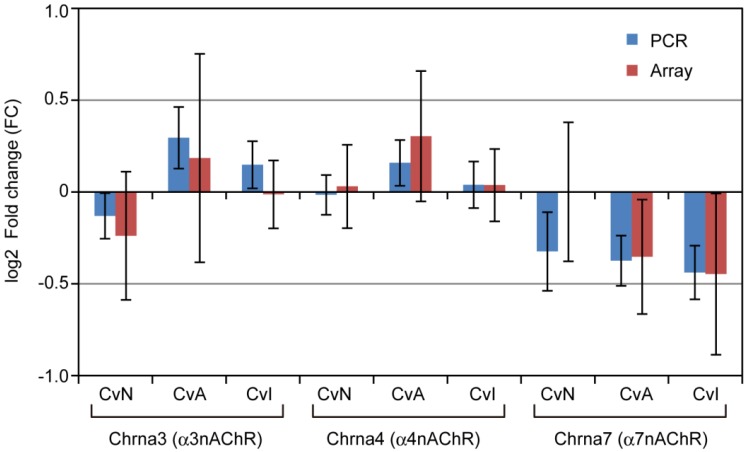
Relative mRNA expression of three nAChRs in cerebellar cultures exposed to nicotine, acetamiprid or imidacloprid for 14 days, at 16 days in vitro, versus control, measured using qRT-PCR and microarray. Error bars represent standard error of six independent experiments. *T*-tests showed no significant differences in expression between PCR and microarray, or between control versus nicotine (CvN), acetamiprid (CvA), or imidacloprid (CvI).

To confirm the expression of nAChRs in the treated cultures at 16 DIV, mRNA expression of Chrna3, Chrna4, and Chrna7 (coding for α3, α4, and α7 nAChRs, respectively) was determined using qRT-PCR. Figure 6 shows their relative expression as measured by PCR and microarray. Expression of these mRNAs after exposure to NIC, ACE or IMI was very similar to that in the control group and their FCs were log_2_ −0.44 to log_2_ 0.29.

### 3.5. Bioinformatics Analysis of DE Genes for CvN, CvA, and CvI

**Figure 7:**
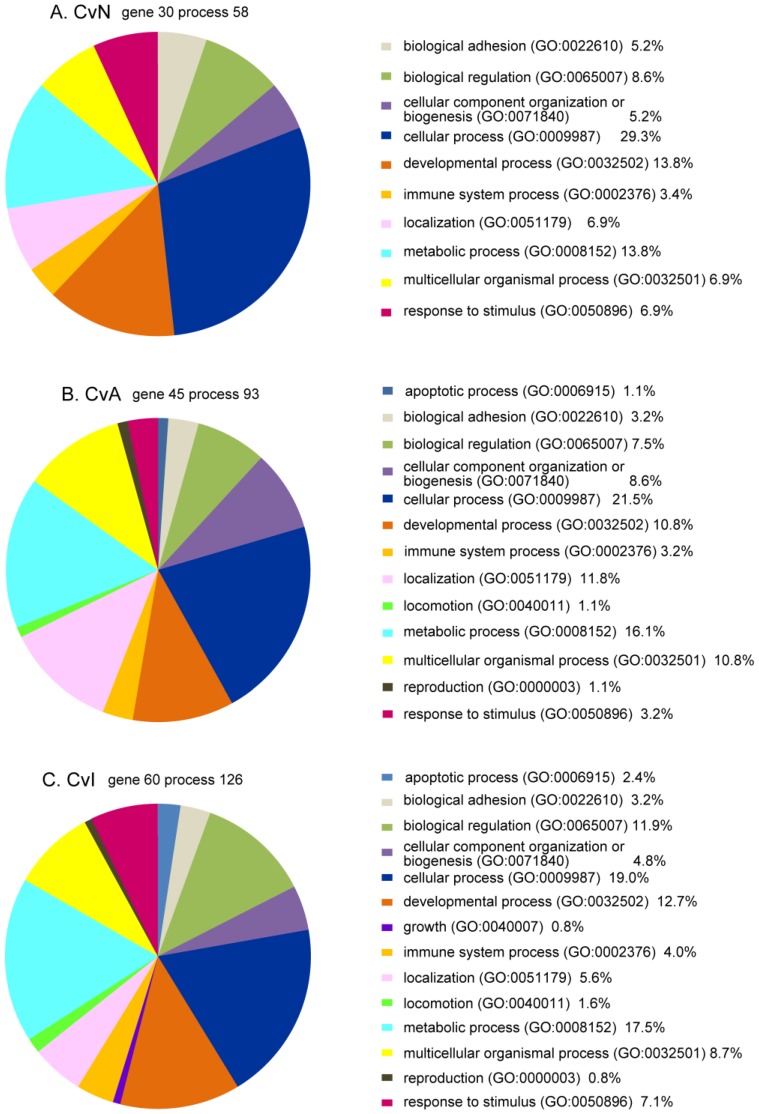
Gene ontology (GO) biological process analyses of differentially expressed (DE) genes for control versus nicotine (CvN), acetamiprid (CvA), or imidacloprid (CvI) in pie charts **A**, **B**, and **C**, respectively. Each gene list was applied to PANTHER software. GO biological processes are shown in pie charts, CvN (**A**), CvA (**B**), and CvI (**C**). A number of genes were not recognized by the PANTHER system, including Ndufaf2 and Sdr42e2 (common to all three lists); Car3 and LOC317356 (CvN); LOC100363332 (CvA); Cyp4a1, Klk1c7, LOC100364862, LOC100912563, and LOC294497 (CvI).

First we applied the PANTHER Classification System to the three group lists of DE genes to gain the overview of the functions of these genes (Figure 7). Most patterns were similar between the three groups and comprised 10 categories: “cellular process”, “metabolic process”, “developmental process”, “biological regulation”, “localization”, “multicellular organismal process”, “response to stimulus”, “biological adhesion”, “cellular component organization or biogenesis”, and “immune system process”. The three GO categories of biological processes (“apoptotic process”, “locomotion”, and “reproduction”) were common to CvA and CvI. Only “growth” was unique to CvI (Figure 7). Details of PANTHER classifications are shown in supplemental Table S3.

We next applied the DE gene lists (CvN, CvA, and CvI), with their FCs and p values to the MetaCore system, and performed bioinformatics enrichment analysis on these data, which revealed 10 lists of significant-scoring GO processes and biomarkers of diseases (Table 5, Table 6 and Table 7). Genes for two types of calcium channels, Cacna1h (voltage-dependent T-type calcium channel subunit α-1H) and Cacng1 (voltage-dependent calcium channel γ-1 subunit) showed differential expression in all three groups and frequently included various categories in related genes, such as “calcium or divalent ion transport” (Table 5, Table 6 and Table 7, GO processes) and “epilepsy”, “brain diseases”, and “seizures” (Table 5, Table 6 and Table 7, diseases). Also, F2rl2 (coagulation factor II (thrombin) receptor-like 2), alias Par3 (proteinase-activated receptor 3) and belonging to a multifunctional subfamily of G-protein coupled receptors (GPCRs), was a DE gene common to all three groups and related to multiple categories, such as “ion transport”, “brain diseases”, “autistic disorder”, and “child development disorders” (Table 5, Table 6 and Table 7). DE genes unique to each group were also related to important categories. These included Ihh (Indian hedgehog; CvN), related to “cell–cell adhesion” and “brain diseases” (Table 5), Hrh2 (histamine receptor H2; CvA), and Htr2c (serotonin receptor 2C; CvI), both related to important categories of GO processes and diseases (Table 6 and Table 7).

Next, we analyzed three groups of DE gene lists by MetaCore’s Compare Experiments Workflow, which is a tool that can be used for comparing experimental data by analyzing their intersections in terms of their mappings onto MetaCore’s various ontologies, including GO processes and diseases. The comparison analysis results of three DE gene lists in CvN, CvA, and CvI revealed significant GO processes and diseases categories (Table 8). The GO processes were associated with “calcium ion transport”, “divalent metal ion transport”, “divalent inorganic cation transport”, etc., most of which were correlated to common DE genes including calcium channels (Cacna1h and Cacng1) and GPCRs (F2rl2). The listed diseases are “epilepsy”, “seizures”, “autistic disorder”, “pervasive child development disorders”, and “mental disorders diagnosed in childhood”, most of which were also correlated with calcium channels and GPCRs, same as GO processes.

## 4. Discussion

This is the first in vitro investigation of transcriptome changes in rat neonatal cerebellar neuron-enriched cultures following long-term exposure (14 days) to NIC and the neonicotinoids ACE and IMI, which are widely used in pesticides. Our results indicate that alterations in transcriptome profiles occur after exposure to these agents.

The DE gene lists of the CvN, CvA, and CvI comparisons included genes unique to each comparison and those genes common to two or more comparisons and support our previous data regarding NIC-like effects of ACE and IMI [19]. Neonicotinoids and NIC all excite small cerebellar neurons, but the firing patterns, firing peaks, and excited cell numbers differ between the different agents.

In the DE genes common to the three groups, those for two types of calcium channels, Cacna1h and Cacng1, are important for neuronal activity. The Cacna1h gene is registered as a strong candidate in the autism-related gene database, SFARI [39]. Mutations in this gene are also found in patients with epilepsy [40,41]. In cerebellar Purkinje cells, parallel fiber excitatory postsynaptic potentials are regulated by K channels and the Cav3.2 (Cacna1h) calcium channel [42]. The Cacng1 gene expressed in human fetal and adult brain [43] and developing mouse cerebellum [44]. A mutation of this gene was also proposed as a candidate for epilepsy [45] and its expression in hippocampal CA1 is altered in drug-induced epilepsy [46]. Another common DE gene, F2rl2 (Par3) belongs to an important subfamily of GPCRs, that are correlated with various phases of neurodevelopment [47,48,49,50], including axon-dendrite differentiation [51]. The remaining DE genes common to the three experimental groups were Cramp1l (alias: hematological and neurological expressed 1-like protein), which is a DNA and chromatin binding protein; Tada2b, a transcriptional adaptor protein; Lmod3, a fetally expressed member of the leiomodin family, which increases actin polymerization rate; Ndufaf2, which is NADH:ubiquinone oxidoreductase complex assembly factor 2; Sdr42e2, one of the short-chain dehydrogenases/reductases; and Unc45b, which acts as a co-chaperone for HSP90 and is required for the proper folding of the myosin motor domain [52]. These genes all have basic proliferation or differentiation functions, and expression of each was confirmed in the developing mouse cerebellum database [44] or the human brain database, GTEx Portal [53].

NIC, ACE, and IMI appeared to cause a slight disturbance in the dendritic arborization of Purkinje cells (Figure 2). This disturbance might be correlated to altered expression of the nine genes. Indeed, F2rl2 (Par3) regulates axon and dendrite differentiation [51], and its disruption might affect this important developmental stage. Further pharmacological and mechanistic investigations are needed to identify the role of these DE genes in the development of Purkinje (and other) cells. Expression of Calb1 (calbindin D28k) was very similar between control cultures and each experimental group in the microarrays.

In the SFARI autism database, the following DE genes were registered: Celf6, which codes for a transcription associated protein and was common to CvN and CvI; Magel2 (melanoma antigen family L2), unique to CvI; and Ampd1 (adenosine monophosphate deaminase), also unique to CvI. Three types of CvN unique genes were registered in the autism KB database [54,55]: Cadm3 (cell adhesion molecule), Gpr83 (G protein-coupled receptor), and Acta1 (actin α1). Four CvA-unique genes were registered in the same database: Napb (related to amyotrophy), Rasl10b, Iqcf1, and Myog (transcription factors). Finally, three CvI-unique are also registered in the Autism KB database: Htr2c, Rbfox2 (an RNA binding protein), and Txk (a tyrosine kinase). The functions and correlations of these disorder-related genes are not yet clear, but given the developmental nature of autism, they may be involved in brain development.

The PANTHER ontology analysis of the three DE gene lists (CvN, CvA, CvI) showed similar and fundamental gene categories for brain development (Figure 7 and supplemental Table S3). Independent analysis of each DE gene list using MetaCore indicated similarities and differences in significant categories of GO processes and diseases (Table 5, Table 6 and Table 7). In the process category, all three experimental groups included “calcium ion transport”, which is essential for neuronal activity. Within the diseases category, “epilepsy”, “autistic disorders”, and “child development disorders, pervasive” were common to the three groups. Moreover, comparing analyses of all three groups confirmed these results (Table 8), and other essential GO process categories (“cellular response to potassium ion”, “metal ion transport”, thrombin receptor signaling”, “positive regulation of exocytosis”, “membrane depolarization”) and disease categories (“mental disorders diagnosed in childhood”, “mitochondrial complex 1 deficiency”, “delirium”, “neurologic manifestations”) are listed with significant p values.

In each enrichment analysis, two GO processes (“glucocorticoid biosynthetic process”, “cellular component morphogenesis”) were common to CvN and CvA, and two diseases (“Tourette syndrome”, “neurotoxicity syndromes”) were common to CvA and CvI. Other disease categories, unique to each group, included “movement disorders” (CvN), “communication disorders” (CvA), and “dyskinesia” (CvI), etc. These analyses suggest that chronic exposure to NIC, ACE or IMI might alter gene expression profiles, which are important for normal brain development. It will be necessary to confirm this in future studies.

A limitation of the present study was that we could not determine whether the altered gene expression profiles were caused by the direct action of NIC, ACE and IMI on nAChRs. However, we did confirm the mRNA expression of α3, α4, and α7 nAChR subunits, both here (Figure 6) and in our previous study [19]. Our previous report indicated that NIC-, ACE-, or IMI-induced Ca^2+^-influx in cerebellar neurons was inhibited by the specific nAChR antagonists dihydro-β-erythroidine (α4β2 specific) and α-bungarotoxin (α7 specific), which suggests that NIC and neonicotinoids act directly at these receptors. Furthermore, we cannot rule out the possibility that some toxic metabolites of ACE or IMI are produced during cultivation, and both the agents and their metabolites might act at these receptors.

Other reports have suggested that neonicotinoids have direct actions on mammalian nAChRs. IMI and clothianidin directly activate human α4β2 nAChRs [9], and modify the receptor’s responses to ACh, even at a concentration below the activation threshold of the receptors.

ACE and IMI have some potency at mammalian α3 and α4β2 nAChRs [3]. Although the authors concluded that IMI and ACE were inactive at α7 nAChRs, their data indicate that ACE and IMI actually inhibited the binding of α-bungarotoxin to rat or human α7 nAChRs. Moreover, α-bungarotoxin antagonized IMI-induced activation of stellate cells of the mouse cochlear nucleus, which suggests that IMI binds to mouse α7 or α9α10 nAChRs [56] and IMI activated mammalian α7 nAChRs expressed in Xenopus oocytes [57]. In excitation assays of chicken α7 nAChRs, two neonicotinoids IMI and thiacloprid actually activated the receptors, even though the excitation peaks were lower than those by ACh or NIC [58]. Those authors expected similar results with human α7, because of its similarity to the avian α7 (93.8% in the extracellular agonist binding regions). Together, these data support the notion that IMI and ACE bind to mammalian α7 nAChRs.

NIC has a lower affinity for α7 nAChRs than heteromeric nAChRs [59]. However, many reports have indicated that NIC causes various effects via α7 nAChRs, even at a low concentration [60,61]. In the brain, the α7 nAChR plays important functions; synaptic plasticity, regulation of neuronal growth, differentiation, and enhancing memory and cognition [62,63,64]. Furthermore, α7 nAChR dysfunction is correlated to the symptoms and etiology of Alzheimer disease [65,66] and schizophrenia [21,67].

ACh and other ligands often cause desensitization of nAChRs, including α4β2 and α7, even concentrations below the receptor’s activation threshold [25,68]. In addition, many factors change the kinetics of receptor desensitization, including subunit composition, temperature, post-translational modification, types of ligand, duration of ligand exposure, and interactions with modulator proteins [69,70,71]. Together, this suggests that the transcriptome profile alterations induced by NIC, ACE, and IMI might be caused by activation and/or desensitization of nAChRs.

In the mammalian nervous system, nAChRs are expressed in glia as well as neurons. For example, α7 subtypes are expressed in astrocytes [72] and microglia [73], and α3, α4, α5, α7, β2, and β4 subtypes are found in oligodendrocyte precursor cells [74]. Such reports, together with the present results, suggest that these glial nAChRs have important regulatory functions that can be disrupted by NIC, ACE, and IMI.

Recently, Meijer et al. reported that several pesticides disturbed KCl-induced Ca^2+^-influx via voltage-gated calcium channels in PC12 or primary cortical cells, but that IMI and carbaryl did not [75,76]. In contrast, our previous report indicated that IMI disturbed KCl-induced Ca^2+^-influx after nAChR activation in 33-40% of rat cerebellar neurons. This discrepancy may be a factor of the time course examined, or differences in the number, subclass, or localization of nAChRs in PC12 and primary cortical cells. Alternatively, the difference may be due to the fact that IMI induced Ca^2+^-influx in small proportion of rat cerebellar neurons, whereas KCl induced significant Ca^2+^-influx in the majority of PC12 cells and also our cerebellar neurons.

To date, there has been no definitive evidence for mammalian developmental neurotoxicity caused by neonicotinoids [77], because of the widely held view that they have a low affinity for mammalian nAChRs, penetrate poorly into the mammalian brain through the BBB, and have a lack of effects in common with NIC. However, increasing evidence to the contrary [11,12,13,14,15,16,17,18,19], and consideration of some neonicotinoid metabolites showing high affinities for mammalian nAChRs [3], suggests that neonicotinoids have potential developmental neurotoxicity.

In utero exposure to NIC in combination with an organophosphate produces significant sensorimotor deficits in rat offspring [78]. In 2012, the American Academy of Pediatrics stated that “epidemiologic evidence demonstrates associations between early life exposure to pesticides and pediatric cancers, decreased cognitive function, and behavioral problems [79],” and “children’s exposures to pesticides should be limited as much as possible [80]”. This statement affirms the neurotoxicity of organophosphate pesticides, which disturb the acetylcholinesterase and cholinergic systems. Actions of NIC and neonicotinoids on nAChRs may increase the cholinergic neurotoxicity of organophosphates.

In a recent report, three major classes of pesticides were shown to be commonly present in urine samples from 3-year-old children in Japan (*n* = 223) [81]. In total, 79.8% of the samples were positive for at least one neonicotinoid (median 3.97 nM and maximum 308.18 nM), and 100% were positive for at least one organophosphate metabolite (median 211.45 nM and maximum 2232.78 nM) and pyrethroid metabolites, 3-PBA (median 1.01 μg/L and maximum 25.29 μg/L). Studies such as this highlight the urgency for investigations into the effects of these neonicotinoids and other pesticides on human health. Although the pesticide concentrations were within the acceptable daily intake, the pesticide registration system in Japan does not include developmental neurotoxicity tests, and combined effects of various pesticides have not yet been examined.

There are several limitations to this study; for example, we examined only one brain region. In several preliminary experiments, we exposed cultured cells from the cerebellum, hippocampus, and cerebral cortex to 1~10 μM NIC, ACE, or IMI at 10 DIV, which resulted in variable transcriptome disturbances, not all of which were statistically significant (not shown). Moreover, we did not examine the protein expressions of DE genes. Despite these limitations, our study suggests that neonicotinoids might affect mammalian brain development. Further research, including dose-dependency and time course studies, functional assays (e.g., Ca2^+^ imaging), protein expression measurements, and in vivo experiments, are needed to define the effects of neonicotinoids on the developing mammalian brain.

## 5. Conclusions

Chronic low-dose exposure of the neonicotinoids ACE, IMI can induce certain alterations in the transcriptome of the developing rodent brain. Several genes known to be essential for brain development were up or downregulated after exposure of cerebellar cultures to NIC, ACE, and IMI, including two types of calcium channels (Cacna1h and Cacng1) and a subfamily of GPCRs (F2rl2/Par3). Bioinformatic analysis suggested a correlation between these transcriptome alterations and developmental disorders such as epilepsy and autistic disorders. In addition, immunocytochemistry in Purkinje cells revealed that chronic exposure to NIC, ACE, or IMI induced small disturbances in dendritic arborization that might correlate to the observed transcriptome alterations. Taken together, our results indicate that neonicotinoids used as pesticides may have some adverse effects on the developing mammalian brain. Even though the affinities of IMI and ACE for mammalian nAChRs are lower than to those for insect nAChRs, the effects of neonicotinoids on the developing brain should be carefully investigated.

## Figures and Tables

**Table 1:** DE genes common to two or three pairs of CvN, CvA, and CvI.

Gene Name	Probe Name ^a^	Description		CvN Log_2_FC, *p, q*	CvA Log_2_FC, *p, q*	CvI Log_2_FC, *p, q*
Common to CvN, CvA, CvI						
Cacna1h	A_64_P067366	calcium channel, voltage-dependent, T type, alpha 1H subunit	up	0.74, 2.8 ×10^−2^, 1.3 × 10^−3^	0.71, 2.8 × 10^−^^2^, 1.6 × 10^−^^3^	0.64, 4.6 × 10^−^^2^, 1.8 × 10^−3^
Cramp1l	A_64_P047944	PREDICTED: Crm, cramped-like (Drosophila)	up	1.22, 4.6 × 10^−^^2^, 1.3 × 10^−3^	0.73, 2.8 × 10^−^^2^, 1.6 × 10^−3^	0.69, 2.8 × 10^−^^2^, 1.8 × 10^−3^
F2rl2	A_44_P306344	coagulation factor II (thrombin) receptor-like 2	up	1.02, 4.6 × 10^−^^2^, 1.3 × 10^−3^	0.89, 2.8 × 10^−^^2^, 1.6 × 10^−3^	0.86, 2.8 × 10^−^^2^, 1.8 × 10^−3^
Tada2b	A_44_P328340	transcriptional adaptor 2B	up	0.66, 2.8 × 10^−^^2^, 1.3 × 10^−3^	1.19, 2.8 × 10^−^^2^, 1.6 × 10^−3^	1.05, 2.8 × 10^−^^2^, 1.8 × 10^−3^
Cacng1	A_64_P064820	calcium channel, voltage-dependent, gamma subunit 1	down	−1.08, 2.8 × 10^−^^2^, 1.3 × 10^−3^	−0.99, 2.8 × 10^−^^2^, 1.6 × 10^−3^	−1.04, 2.8 × 10^−^^2^, 1.8 × 10^−3^
Lmod3	A_64_P103610	leiomodin 3 (fetal)	down	−2.01, 4.6 × 10^−^^2^, 1.3 × 10^−3^	−1.75, 4.6 × 10^−^^2^, 1.6 × 10^−3^	−1.65, 4.6 × 10^−^^2^, 1.8 × 10^−3^
Ndufaf2	A_43_P22034	PREDICTED: NADH dehydrogenase (ubiquinone) complex I, assembly factor 2	down	−0.79, 2.8 × 10^−^^2^, 1.3 × 10^−3^	−0.69, 2.8 × 10^−^^2^, 1.6 × 10^−3^	−0.80, 2.8 × 10^−^^2^, 1.8 × 10^−3^
Sdr42e2	A_64_P077921	PREDICTED: short chain dehydrogenase/reductase family 42E, member 2	down	−0.78, 4.6 × 10^−^^2^ 1.3 × 10^−3^	−0.86, 4.6 × 10^−^^2^, 1.6 × 10^−3^	−0.81, 2.8 × 10^−^^2^, 1.8 × 10^−3^
Unc45b	A_42_P579305	unc-45 homolog B (C. elegans)	down	−0.75, 4.6 × 10^−^^2^, 1.3 × 10^−3^	−0.84, 2.8 × 10^−^^2^, 1.6 × 10^−3^	−0.65, 2.8 × 10^−^^2^, 1.8 × 10^−3^
Common to CvN, CvA						
Mcmdc2	A_64_P000381	minichromosome maintenance domain containing 2	up	0.73, 2.8 × 10^−^^2^, 1.3 × 10^−3^	1.17, 4.6 × 10^−^^2^, 1.6 × 10^−3^	0.23, 4.6 × 10^−1^, 5.4 × 10^−3^
Actrt2	A_44_P105554	actin-related protein T2	down	−0.93, 2.8 × 10^−^^2^, 1.3 × 10^−3^	−1.08, 4.6 × 10^−^^2^, 1.6 × 10^−3^	−0.68, 7.5 × 10^−1^, 7.0 × 10^−3^
Ankrd60	A_64_P002697	Protein Ankrd60	down	−0.97, 4.6 × 10^−^^2^, 1.3 × 10^−3^	−0.88, 2.8 × 10^−^^2^, 1.6 × 10^−3^	−0.87, 1.2 × 10^−1^, 2.7 × 10^−3^
Common to CvN, CvI						
Celf6	A_44_P527809	CUGBP, Elav-like family member 6	up	0.72, 4.6 × 10^−^^2^, 1.3 × 10^−3^	−0.11, 3.5 × 10^−1^, 3.8 × 10^−3^	0.63, 4.6 × 10^−^^2^, 1.8 × 10^−3^
Pcdhgb7	A_64_P079069	protocadherin gamma subfamily B, 7	up	0.95, 4.6 × 10^−^^2^, 1.3 × 10^−3^	0.27, 4.6 × 10^−^^2^, 1.6 × 10^−3^	0.61, 4.6 × 10^−^^2^, 1.8 × 10^−3^
Cyp17a1	A_44_P508386	cytochrome P450, family 17, subfamily a, polypeptide 1	down	−0.59, 2.8 × 10^−^^2^, 1.3×10^−3^	−0.58, 2.8 × 10^−^^2^, 1.6 × 10^−3^	−0.73, 4.6 x 10^−^^2^, 1.8 × 10^−3^
Mypn	A_44_P159259	myopalladin	down	−0.66, 2.8 × 10^−^^2^, 1.3 × 10^−3^	−0.50, 2.5 × 10^−1^, 3.3 × 10^−3^	−0.71, 2.8 × 10^−^^2^, 1.8 × 10^−3^
Zp3	A_64_P044241	zona pellucida glycoprotein 3 (sperm receptor)	down	−0.82, 2.8 × 10^−^^2^, 1.3 × 10^−3^	−0.54, 4.6 × 10^−1^, 4.4 × 10^−3^	−1.08, 2.8 × 10^−^^2^, 1.8 × 10^−3^
Common to CvA, CvI						
Lyn	A_64_P048980	v-yes-1 Yamaguchi sarcoma viral related oncogene homolog	up	0.25, 1.2 × 10^−1^, 1.9 × 10^−3^	1.12, 2.8 × 10^−^^2^, 1.6 × 10^−3^	0.73, 2.8 × 10^−^^2^, 1.8 × 10^−3^
Hspb7	A_43_P15812	heat shock protein family, member 7 (cardiovascular)	down	−0.26, 7.5 × 10^−1^, 4.8 × 10^−3^	−1.07, 2.8 × 10^−^^2^, 1.6 × 10^−3^	−1.30, 4.6 × 10^−^^2^, 1.8 × 10^−3^
Mb	A_42_P765066	myoglobin	down	−0.72, 1.2 × 10^−1^, 1.9 × 10^−3^	−1.1, 2.8 × 10^−^^2^, 1.6 × 10^−3^	−0.82, 2.8 × 10^−^^2^, 1.8 × 10^−3^
Tnni2	A_42_P718022	troponin I type 2 (skeletal, fast)	down	−1.00, 1.2 × 10^−1^, 1.9 × 10^−3^	−0.94, 2.8 × 10^−^^2^, 1.6 × 10^−3^	−0.85, 4.6 × 10^−^^2^, 1.8 × 10^−3^

^a^ Probe names are Agilent ID numbers of Rat Gene Expression ver.3 Microarray. DE: differentially expressed; CvN: control versus NIC; CvA: control versus ACE; CvI: control versus IMI; FC: fold change; positive FC = upregulation versus control; negative FC = downregulation versus control; *p*: *p*-value; *q*: false discovery rate; gray column: less than FC ≥ 1.5 (log_2_ 0.585) or greater than *p* < 0.05; FC, *p*, and *q* values were calculated from microarray data of six independent experiments.

**Table 2:** DE genes unique to CvN.

Gene Name	Probe Name ^a^	Description		Log_2_FC, *p*, *q*
Atf7	A_64_P108639	activating transcription factor 7	up	0.66, 4.6 × 10^−^^2^, 1.3 × 10^−3^
Cadm3	A_44_P454645	cell adhesion molecule 3	up	0.61, 2.8 × 10^−^^2^, 1.3 × 10^−3^
Cd86	A_44_P222166	CD86 molecule	up	0.60, 4.6 × 10^−^^2^, 1.3 × 10^−3^
Gpr83	A_44_P269457	G protein-coupled receptor 83	up	0.75, 2.8 × 10^−^^2^, 1.3 × 10^−3^
Gramd4	A_64_P143562	PREDICTED:GRAM domain containing 4	up	0.71, 2.8 × 10^−^^2^, 1.3 × 10^−3^
Ihh	A_43_P15154	Indian hedgehog	up	0.91, 4.6 × 10^−^^2^, 1.3 × 10^−3^
Kcnq5	A_64_P149659	potassium voltage-gated channel, KQT-like subfamily, member 5	up	1.86, 4.6 × 10^−^^2^, 1.3 × 10^−3^
Klhl18	A_64_P100973	Protein Klhl18	up	0.62, 2.8 × 10^−^^2^, 1.3 × 10^−3^
LOC317356	A_64_P009658	PREDICTED: protocadherin alpha-13-like	up	1.07, 4.6 × 10^−^^2^, 1.3 × 10^−3^
Nt5dc1	A_44_P440988	5’-nucleotidase domain containing 1	up	0.66, 2.8 × 10^−^^2^, 1.3 × 10^−3^
Vsig2	A_64_P117949	V-set and immunoglobulin domain containing 2	up	0.64, 4.6 × 10^−^^2^, 1.3 × 10^−3^
Acta1	A_44_P255236	actin, alpha 1, skeletal muscle (Acta1)	down	−1.25, 4.6 × 10^−^^2^, 1.3 × 10^−3^
Car3	A_44_P244851	carbonic anhydrase 3	down	−0.75, 4.6 × 10^−^^2^, 1.3 × 10^−3^
Ms4a12	A_64_P078240	PREDICTED: membrane-spanning 4-domains, subfamily A, member 12	down	−0.59, 2.8 × 10^−^^2^, 1.3 × 10^−3^
Nhlh2	A_64_P157873	nescient helix loop helix 2	down	−0.68, 4.6 × 10^−^^2^, 1.3 × 10^−3^
Olr1051	A_64_P089733	olfactory receptor 1051	down	−1.27, 2.8 × 10^−^^2^, 1.3 × 10^−3^
Phldb3	A_42_P599116	pleckstrin homology-like domain, family B, member 3	down	−0.84, 4.6 × 10^−^^2^, 1.3 × 10^−3^

^a^ Probe names are Agilent ID numbers of Rat Gene Expression ver.3 Microarray. DE: differentially expressed; CvN: control versus NIC; FC: fold change; positive FC = upregulation; negative FC = downregulation versus control; *p*: *p*-value; *q*: false discovery rate; FC, *p*, and *q* values were calculated from microarray data of six independent experiments.

**Table 3:** DE genes unique to CvA.

Gene Name	Probe Name ^a^	Description		Log_2_FC, *p*, *q*
Asap2	A_43_P20879	Protein Asap2	up	0.64, 2.8 × 10^−^^2^, 1.6 × 10^−3^
B3gnt9	A_64_P067868	PREDICTED: UDP-GlcNAc:betaGal beta-1,3-N-acetylglucosaminyltransferase 9	up	1.81, 2.8 × 10^-2^, 1.6 × 10^−3^
Dupd1	A_64_P029907	dual specificity phosphatase and pro isomerase domain containing 1	up	1.84, 2.8 × 10^−^^2^, 1.6 × 10^−3^
Fyb	A_64_P101995	FYN binding protein	up	0.59, 2.8 × 10^−^^2^, 1.6 × 10^−3^
Hrh2	A_64_P030162	histamine receptor H 2	up	1.29, 2.8 × 10^−^^2^, 1.6 × 10^−3^
LOC100363332	A_44_P421727	caspase recruitment domain family, member 11	up	0.71, 4.6 × 10^−^^2^, 1.6 × 10^−3^
LOC499235	A_64_P145473	LRRGT00141; Protein LOC499235	up	0.64, 2.8 × 10^−^^2^, 1.6 × 10^−3^
LOC679149	A_64_P052159	similar to carboxylesterase 2 (intestine, liver)	up	0.75, 2.8 × 10^−^^2^, 1.6 × 10^−3^
Mroh1	A_64_P001622	maestro heat-like repeat family member 1	up	0.60, 2.8 × 10^−^^2^, 1.6 × 10^−3^
Napb	A_44_P333146	N-ethylmaleimide-sensitive factor attachment protein, beta	up	0.66, 2.8 × 10^−^^2^, 1.6 × 10^−3^
Ntn1	A_64_P023527	netrin 1	up	0.69, 2.8 × 10^−^^2^, 1.6 × 10^−3^
Phf21b	A_44_P234547	PHD finger protein 21B	up	0.72, 2.8 × 10^−^^2^, 1.6 × 10^−3^
Plac8	A_44_P212964	placenta-specific 8	up	0.65, 4.6 × 10^−^^2^, 1.6 × 10^−3^
Rasl10b	A_64_P126140	RAS-like, family 10, member B	up	0.68, 2.8 × 10^−^^2^, 1.6 × 10^−3^
Slc45a3	A_43_P10089	solute carrier family 45, member 3	up	1.11, 4.6 × 10^−^^2^, 1.6 × 10^−3^
Zfp498	A_64_P024399	zinc finger protein 498	up	0.66, 4.6 × 10^−^^2^, 1.6 × 10^−3^
Actc1	A_64_P078862	actin, alpha, cardiac muscle 1	down	−1.14, 4.6 × 10^−^^2^, 1.6 × 10^−3^
Dcdc2	A_44_P685554	doublecortin domain containing 2	down	−0.76, 4.6 × 10^−^^2^, 1.6 × 10^−3^
Des	A_44_P384090	desmin	down	−1.64, 2.8 × 10^−^^2^, 1.6 × 10^−3^
Dmbt1	A_64_P237865	deleted in malignant brain tumors 1	down	−0.89, 2.8 × 10^−^^2^, 1.6 × 10^−3^
Hsd3b1	A_64_P123655	hydroxy-delta-5-steroid dehydrogenase, 3 beta- and steroid delta-isomerase 1	down	−0.91, 2.8 × 10^−^^2^, 1.6 × 10^−3^
Iqcf1	A_44_P744940	IQ motif containing F1	down	−0.60, 2.8 × 10^−^^2^, 1.6 × 10^−3^
Lmod2	A_44_P1003728	leiomodin 2 (cardiac)	down	−1.63, 2.8 × 10^−^^2^, 1.6 × 10^−3^
LOC684762	A_42_P755367	PREDICTED: histone H3.2-like	down	−1.49, 4.6 × 10^−^^2^, 1.6 × 10^−3^
Myog	A_64_P056293	myogenin	down	−0.74, 2.8 × 10^−^^2^, 1.6 × 10^−3^
Nags	A_42_P803406	N-acetylglutamate synthase	down	−1.05, 4.6 × 10^−^^2^, 1.6 × 10^−3^
Olr1714	A_64_P043808	olfactory receptor 1714	down	−0.80, 2.8 × 10^−^^2^, 1.6 × 10^−3^
Olr825	A_44_P130360	olfactory receptor 825	down	−0.60, 2.8 × 10^−^^2^, 1.6 × 10^−3^
Olr94	A_44_P285601	olfactory receptor 94	down	−0.70, 4.6 × 10^−^^2^, 1.6 × 10^−3^
Slc5a5	A_64_P080233	solute carrier family 5 (sodium/iodide cotransporter), member 5	down	−0.68, 4.6 × 10^−^^2^, 1.6 × 10^−3^
Slco1b2	A_44_P328097	solute carrier organic anion transporter family, member 1B2	down	−1.41, 2.8 × 10^−^^2^, 1.6 × 10^−3^
Vom1r73	A_64_P097332	vomeronasal 1 receptor 73	down	−0.59, 2.8 × 10^−^^2^, 1.6 × 10^−3^

^a^ Probe names are Agilent ID numbers of Rat Gene Expression ver.3 Microarray. DE: differentially expressed; CvA: control versus ACE; FC: fold change; positive FC = upregulation versus control; negative FC = downregulation versus control; *p*: *p*-value; *q*: false discovery rate; FC, *p*, and *q* values were calculated from microarray data of six independent experiments.

**Table 4:** DE genes unique to CvI.

Gene Symbol	Probe Name ^a^	Description		Log_2_FC, *p*, *q*
Ccdc81	A_44_P499041	coiled-coil domain containing 81	up	0.62, 4.6 × 10^−^^2^, 1.8 × 10^−3^
Cdc6	A_64_P052665	cell division cycle 6	up	0.68, 4.6 × 10^−^^2^, 1.8 × 10^−3^
Crb3	A_44_P1043302	crumbs homolog 3 (Drosophila)	up	0.89, 2.8 × 10^−^^2^, 1.8 × 10^−3^
Cxcl17	A_64_P053431	chemokine (C-X-C motif) ligand 17	up	0.70, 4.6 × 10^−^^2^, 1.8 × 10^−3^
Efna3	A_64_P087380	PREDICTED: ephrin A3 (Efna3)	up	0.70, 2.8 × 10^−^^2^, 1.8 × 10^−3^
Galnt7	A_44_P406169	UDP-N-acetyl-α-D-galactosamine:polypeptide N-acetylgalactosaminyltransferase 7 (GalNAc-T7)	up	0.66, 4.6 × 10^−^^2^, 1.8 × 10^−3^
Grb7	A_64_P135843	growth factor receptor bound protein 7	up	0.77, 4.6 × 10^−^^2^, 1.8 × 10^−3^
Htr2c	A_44_P792784	5-hydroxytryptamine (serotonin) receptor 2C	up	1.61, 2.8 × 10^−^^2^, 1.8 × 10^−3^
Iqsec3	A_64_P037666	IQ motif and Sec7 domain 3	up	0.62, 2.8 × 10^−^^2^, 1.8 × 10^−3^
Lbx1	A_64_P009892	ladybird homeobox 1	up	1.10, 2.8 × 10^−^^2^, 1.8 × 10^−3^
Magel2	A_64_P065185	PREDICTED: MAGE-like 2	up	0.63, 2.8 × 10^−^^2^, 1.8 × 10^−3^
Mybpc2	A_44_P201295	myosin binding protein C, fast-type	up	0.64, 2.8 × 10^−^^2^, 1.8 × 10^−3^
Oasl	A_44_P492025	2’-5’-oligoadenylate synthetase-like	up	0.77, 4.6 × 10^−^^2^, 1.8 × 10^−3^
Plekha2	A_64_P136831	PREDICTED: pleckstrin homology domain-containing, family A (phosphoinositide binding specific) member 2	up	0.75, 4.6 × 10^−^^2^, 1.8 × 10^−3^
Rbfox2	A_44_P403410	RNA binding protein, fox-1 homolog (C. elegans) 2	up	0.67, 2.8 × 10^−^^2^, 1.8 × 10^−3^
S100pbp	A_64_P084038	S100P binding protein	up	0.61, 2.8 × 10^−^^2^, 1.8 × 10^−3^
Slc2a3	A_44_P292510	solute carrier family 2 (facilitated glucose transporter), member 3	up	0.74, 4.6 × 10^−^^2^, 1.8 × 10^−3^
Syne4	A_64_P006878	spectrin repeat containing, nuclear envelope family member 4	up	1.01, 4.6 × 10^-2^, 1.8 × 10^−3^
Tacr3	A_43_P11794	tachykinin receptor 3	up	0.86, 2.8 × 10^−^^2^, 1.8 × 10^−3^
Txk	A_64_P100138	TXK tyrosine kinase	up	0.71, 2.8 × 10^−^^2^, 1.8 × 10^−3^
Amer3	A_64_P009815	PREDICTED:APC membrane recruitment protein 3	down	−0.72, 4.6 × 10^−^^2^, 1.8 × 10^−3^
Ampd1	A_44_P276087	adenosine monophosphate deaminase 1	down	−0.83, 2.8 × 10^−^^2^, 1.8 × 10^−3^
Bmp8b	A_64_P039949	PREDICTED: bone morphogenetic protein 8b	down	−0.67, 4.6 × 10^−^^2^, 1.8 × 10^−3^
C2cd4c	A_64_P000514	C2 calcium-dependent domain containing 4C	down	−0.64, 2.8 × 10^−^^2^, 1.8 × 10^−3^
Cyp4a1	A_64_P040870	cytochrome P450, family 4, subfamily a, polypeptide 1	down	−0.65, 4.6 × 10^−^^2^, 1.8 × 10^−3^
Egr3	A_64_P128219	early growth response 3	down	−0.77, 4.6 × 10^−^^2^, 1.8 × 10^−3^
Gapt	A_64_P093787	Grb2-binding adaptor protein, transmembrane	down	−0.71, 2.8 × 10^−^^2^, 1.8 × 10^−3^
Hao1	A_44_P340172	hydroxyacid oxidase (glycolate oxidase) 1	down	−0.80, 2.8 × 10^−^^2^, 1.8 × 10^−3^
Hfe2	A_44_P140248	hemochromatosis type 2 (juvenile)	down	−1.67, 4.6 × 10^−^^2^, 1.8 × 10^−3^
Hlcs	A_64_P059834	PREDICTED: holocarboxylase synthetase (biotin-(proprionyl-CoA-carboxylase (ATP-hydrolysing)) ligase)	down	−0.59, 2.8 × 10^−^^2^, 1.8 × 10^−3^
Itpripl1	A_44_P943876	inositol 1,4,5-trisphosphate receptor interacting protein-like 1	down	−0.79, 4.6 × 10^−^^2^, 1.8 × 10^−3^
Klk1c7	A_44_P399414	kallikrein 1-related peptidase C7	down	−1.22, 4.6 × 10^−^^2^, 1.8 × 10^−3^
Krt4	A_64_P105389	keratin 4	down	−0.83, 2.8 × 10^−^^2^, 1.8 × 10^−3^
Krt5	A_44_P461130	keratin 5	down	−0.66, 2.8 × 10^−^^2^, 1.8 × 10^−3^
LOC100364862	A_64_P078448	PREDICTED: hypothetical protein LOC100364862	down	−0.88, 4.6 × 10^−^^2^, 1.8 × 10^−3^
LOC100912563	A_64_P007208	PREDICTED: insulinoma-associated protein 1-like	down	−1.19, 2.8 × 10^−^^2^, 1.8 × 10^−3^
LOC294497	A_64_P000312	similar to double homeobox, 4; double homeobox protein 4	down	−0.61, 2.8 × 10^−^^2^, 1.8 × 10^−3^
LOC685406	A_64_P009947	LRRGT00062	down	−0.76, 2.8 × 10^−^^2^, 1.8 × 10^−3^
Mrgprb4	A_64_P090883	MAS-related GPR, member B4	down	−0.65, 2.8 × 10^−^^2^, 1.8 × 10^−3^
Neurog3	A_44_P353797	neurogenin 3	down	−0.75, 4.6 × 10^−^^2^, 1.8 × 10^−3^
Pitx3	A_64_P072653	paired-like homeodomain 3	down	−0.76, 4.6 × 10^−^^2^, 1.8 × 10^−3^
Prr16	A_64_P026928	proline rich 16	down	−0.89, 2.8 × 10^−^^2^, 1.8 × 10^−3^
RGD1561114	A_64_P012158	PREDICTED: ral guanine nucleotide dissociation stimulator-like	down	−0.62, 4.6 × 10^−^^2^, 1.8 × 10^−3^
RGD1564095	A_64_P043524	PREDICTED: 60S acidic ribosomal protein P2-like	down	−0.88, 2.8 × 10^−^^2^, 1.8 × 10^−3^
RGD1564571	A_64_P159245	PREDICTED: CD209 antigen-like protein A-like	down	−0.61, 2.8 × 10^−^^2^, 1.8 × 10^−3^
RGD1566248	A_64_P157099	PREDICTED: necdin-like	down	−1.17, 4.6 × 10^−^^2^, 1.8 × 10^−3^
Scnn1b	A_64_P068913	sodium channel, non-voltage-gated 1, beta subunit	down	−1.02, 2.8 × 10^−^^2^, 1.8 × 10^−3^
Slc38a8	A_64_P056897	solute carrier family 38, member 8	down	−0.77, 2.8 × 10^−^^2^, 1.8 × 10^−3^
Tmem52b	A_64_P114148	transmembrane protein 52B	down	−0.70, 4.6 × 10^−^^2^, 1.8 × 10^−3^

^a^ Probe names are Agilent ID numbers of Rat Gene Expression ver.3 Microarray. DE: differentially expressed; CvI: control versus IMI; FC: fold change; positive FC = upregulation versus control; negative FC = downregulation versus control; *p*: *p*-value; *q*: false discovery rate; FC, *p*, and *q* values were calculated from microarray data of six independent experiments.

**Table 5:** MetaCore enrichment analysis of DE genes in CvN.

Enrichment Categories	*p*-Value	FDR	Related Genes
GO processes			
Calcium ion transmembrane transport	7.3 × 10^−7^	1.5 × 10^−4^	Cacna1h, Cacng1, Ms4a12, Zp3
Calcium ion transport	8.2 × 10^−7^	1.6 × 10^−4^	Cacna1h, Cacng1, Ms4a12, Zp3, F2rl2
Cellular component assembly involved in morphogenesis	1.3 × 10^−6^	2.1 × 10^−4^	Lmod3, Acta1, Ihh, Mypn,
Glucocorticoid biosynthetic process	1.4 × 10^−6^	2.1 × 10^−4^	Cacna1h, Cyp17a1
Divalent metal ion transport	2.5 × 10^−6^	3.0 × 10^−4^	Cacna1h, Cacng1, Ms4a12, Zp3, F2rl2
Divalent inorganic cation transport	2.6 × 10^−6^	3.0 × 10^−4^	Cacna1h, Cacng1, Ms4a12, Zp3, F2rl2
Negative regulation of astrocyte differentiation	4.6 × 10^−6^	4.3 × 10^−4^	Atf7, F2rl2
Response to steroid hormone	1.0 × 10^−5^	7.0 × 10^−4^	Acta1, Ihh, Atf7, Cyp17a1, Cacna1h, F2rl2, Gpr83
Cell-cell adhesion	1.1 × 10^−5^	7.1 × 10^−4^	Cd86, Ihh, Pcdhgb7, Atf7, Acta1, F2rl2, Cadm3
Cellular component morphogenesis	1.4 × 10^−5^	8.4 × 10^−4^	Lmod3, Acta1, Ihh, Mypn, Cacna1h, Atf7, F2rl2
Diseases (in biomarkers)			
Epilepsy, absence	8.9 × 10^−5^	4.2 × 10^−3^	Cacna1h, Cacng1
Movement disorders	4.7 × 10^−4^	1.2 × 10^−2^	Acta1, Ihh, Cyp17a1, Atf7, F2rl2
Epilepsy, generalized	5.5 × 10^−4^	1.3 × 10^−2^	Cacna1h, Cacng1
Neurodegenerative diseases	1.4 × 10^−3^	2.0 × 10^−2^	Cd86, Lmod3, Ihh, Cyp17a1, Cacng1, Acta1, Atf7, F2rl2
Brain diseases	1.4 × 10^−3^	2.0 × 10^−2^	Cd86, Lmod3, Ihh, Cyp17a1, Cacng1, Cacna1h, Acta1, Atf7, F2rl2
Demyelinating autoimmune diseases, CNS	1.5 × 10^−3^	2.0 × 10^−2^	Cd86, Ihh, Cacng1, Acta1, F2rl2
Seizures	1.7 × 10^−3^	2.0 × 10^−2^	Cacna1h, Cacng1
Autoimmune diseases of the nervous system	2.0 × 10^−3^	2.3 × 10^−2^	Cd86, Ihh, Cacng1, Acta1, F2rl2
Autistic disorder	2.0 × 10^−3^	2.3 × 10^−2^	Cacna1h, F2rl2
Child development disorders, pervasive	2.1 × 10^−3^	2.3 × 10^−2^	Cacna1h, F2rl2

Significant GO process and disease categories are listed for CvN; 34 DE genes were selected. *p*-value and FDR were calculated by MetaCore software. FDR was calculated using the Benjamini and Hochberg method. DE: differentially expressed; CvN: control versus NIC; FDR: false discovery rate; GO: gene ontology.

**Table 6:** MetaCore enrichment analysis of DE genes in CvA.

Enrichment Categories	*p*-Value	FDR	Related Genes
GO processes			
Negative regulation of eicosanoid secretion	7.5 × 10^−9^	3.0 × 10^−6^	Hrh2, F2rl2
Glucocorticoid biosynthetic process	1.6 × 10^−8^	3.8 × 10^−6^	Cacna1h, Hsd3b1
Negative regulation of fatty acid transport	4.1 × 10^−8^	7.6 × 10^−6^	Hrh2, F2rl2
System process	4.5 × 10^−7^	4.5 × 10^−5^	Actc1, Lmod3, Cacng1, Des, Dcdc2, Tnni2, Hrh2, Myog, Cacna1h, Lmod2, F2rl2, Mb, Rasl10b, Olr1714
Response to hormone	1.1 × 10^−6^	8.5 × 10^−5^	Actc1, Lyn, Slco1b2 Dmbt1, Hsd3b1, Myog, Cacna1h, Hrh2, Myb, F2rl2
Cellular component morphogenesis	1.2 × 10^−6^	8.9 × 10^−5^	Actc1, Cacna1h, Hrh2, F2rl2, Lyn, Dcdc2, Lmod2, Lmod3, Ntn1
Regulation of neurotransmitter secretion	2.5 × 10^−6^	1.5 × 10^−4^	Cacna1h, Cacng1, Hrh2
Calcium ion transmembrane transport	3.6 × 10^−6^	2.0 × 10^−4^	Cacna1h, Cacng1, Hrh2
Positive regulation of gamma-aminobutyric acid secretion	3.9 × 10^−6^	2.1 × 10^−4^	Hrh2, F2rl2
Calcium ion transport	5.1 × 10^−6^	2.5 × 10^−4^	Cacna1h, Cacng1, F2rl2, Hrh2
Diseases (in biomarkers)			
Autistic disorder	1.5 × 10^−5^	8.9 × 10^−4^	Cacna1h, Hrh2, F2rl2
Child development disorders, pervasive	1.6 × 10^−5^	9.0 × 10^−4^	Cacna1h, Hrh2, F2rl2
Communication disorders	6.1 × 10^−5^	2.3 × 10^−3^	Dcdc2, Hrh2
Language disorders	6.1 × 10^−5^	2.3 × 10^−3^	Dcdc2, Hrh2
Mental disorders diagnosed in childhood	8.8 × 10^−5^	3.0 × 10^−3^	Cacna1h, Dcdc2, Hrh2
Tourette syndrome	1.3 × 10^−4^	4.1 × 10^−3^	Hrh2, F2rl2
Tic disorders	1.3 × 10^−4^	4.2 × 10^−3^	Hrh2, F2rl2
Epilepsy, absence	1.7 × 10^−4^	4.8 × 10^−3^	Cacna1h, Cacng1
Neurotoxicity syndromes	2.1 × 10^−4^	4.8 × 10^−3^	Hrh2, F2rl2
Pathological conditions, signs and symptoms	5.6 × 10^−4^	9.1 × 10^−3^	Des, Dcdc2, Ntn1, Cacng1, Cacna1h, Hsd3b1, Tnni2, F2rl2, Mb, Dmbt1, Actc1, Nags, Napb

Significant GO process and disease categories are listed for CvA; 48 DE genes were selected. *p*-value and FDR were calculated using MetaCore software. FDR was calculated using the Benjamini and Hochberg method. DE: differentially expressed; CvA: control versus ACE; FDR: false discovery rate; GO: gene ontology.

**Table 7:** MetaCore enrichment analysis of DE genes in CvI.

Enrichment Categories	*p*-Value	FDR	Related Genes
GO processes			
Positive regulation of phosphatidylinositol biosynthetic process	7.5 × 10^−10^	1.1 × 10^−6^	Htr2c, ZP3
Positive regulation of acetylcholine secretion, neurotransmission	3.5 × 10^−9^	3.3 × 10^−6^	Htr2c, F2rl2, Tacr3
Regulation of dopamine metabolic process	1.2 × 10^−8^	5.7 × 10^−6^	Htr2c, F2rl2, Tacr3
Regulation of catecholamine metabolic process	1.4 × 10^−8^	5.9 × 10^−6^	Htr2c, F2rl2, Tacr3
Positive regulation of gamma-aminobutyric acid secretion	4.9 × 10^−8^	1.6 × 10^−5^	Htr2c, F2rl2, Tacr3
Regulation of acetylcholine secretion, neurotransmission	1.2 × 10^−7^	2.8 × 10^−5^	Htr2c, F2rl2, Tacr3
Calcium ion transport	1.3 × 10^−7^	2.8 × 10^−5^	Cacna1h, Cacng1, Htr2c, F2rl2, Tacr3, ZP3
Positive regulation of synaptic transmission, cholinergic	1.5 × 10^−7^	2.8 × 10^−5^	Htr2c, F2rl2, Tacr3
Regulation of gamma-aminobutyric acid secretion	1.5 × 10^−7^	2.8 × 10^−5^	Htr2c, F2rl2, Tacr3
Cation transport	3.7 × 10^−6^	2.6 × 10^−4^	Cacng1, Htr2c, Lyn, Cacna1h, F2rl2, Tacr3, Slc2a3, Slc38a8, Scnn1b, Zp3
Diseases (in biomarkers)			
Dyskinesia, drug-induced	2.0 × 10^−7^	1.2 × 10^−4^	Htr2c, Cyp17a1
Neurotoxicity syndromes	2.7 × 10^−7^	1.2 × 10^−4^	Htr2c, F2rl2, Tacr3, Cyp17a1
Borderline personality disorder	5.1 × 10^−6^	9.2 × 10^−4^	Htr2c, F2rl2, Tacr3
Mental disorders diagnosed in childhood	1.7 × 10^−5^	1.5 × 10^−3^	Cacna1h, Htr2c, F2rl2, Tacr3
Mutism	1.8 × 10^−5^	1.5 × 10^−3^	Htr2c
Autistic disorder	3.6 × 10^−5^	1.7 × 10^−3^	Cacna1h, Htr2c, F2rl2, Tacr3
Child development disorders, pervasive	3.9 × 10^−5^	1.7 × 10^−3^	Cacna1h, Htr2c, F2rl2, Tacr3
Medulloblastoma	2.1 × 10^−4^	5.6 × 10^−3^	Htr2c, Mypn, Slc38a8, Slc2a3, Cacna1h, Txk, Syne4, F2rl2, Tacr3, Pcdhgb7, Celf6, Hfe2, Efna3, Ampd1
Tourette syndrome	2.2 × 10^−4^	5.7 × 10^−3^	Htr2c, F2rl2, Tacr3
Epilepsy, absence	2.9 × 10^−4^	6.9 × 10^−3^	Cacna1h, Cacng1

Significant GO process and disease categories are listed for CvI; 67 DE genes were selected. *p*-value and FDR are calculated using MetaCore software. FDR was calculated using the Benjamini and Hochberg method. DE: differentially expressed; CvI: control versus IMI; FDR: false discovery rate; GO: gene ontology.

**Table 8:** MetaCore enrichment analysis comparing DE genes in the three groups (CvN, CvA, CvI).

Enrichment Categories	*p*-Value	FDR	Related Common Genes	Other Related Genes
GO processes				
Calcium ion transport	1.4 × 10^−7^	6.9 × 10^−5^	Cacna1h, Cacng1, F2rl2	Hrh2 ^a^, Htr2c ^b^, Zp3 ^b,c^, Tacr3 ^b^ Msa4a12 ^c^
Divalent metal ion transport	3.2 × 10^−7^	1.1 × 10^−4^	Cacna1h, Cacng1, F2rl2	Hrh2, Htr2c, Zp3, Tacr3, Msa4a12
Calcium ion transmembrane transport	1.1 × 10^−6^	3.1 × 10^−4^	Cacna1h, Cacng1	Hrh2, Htr2c, Zp3, Msa4a12
Regulation of ion transmembrane transport	1.9 × 10^−6^	4.2 × 10^−4^	Cacna1h, Cacng1, F2rl2	Hrh2, Htr2c, Zp3, Tacr3, Kcnq5 ^c^
Cellular response to potassium ion	1.1 × 10^−5^	1.1 × 10^−3^	Cacna1h	
Metal ion transport	1.2 × 10^−5^	1.1 × 10^−3^	Cacna1h, Cacng1, F2rl2	Hrh2, Htr2c, Zp3, Tacr3, Kcnq5, Msa4a12, Scnn1b ^b^, Slc38a8 ^b^, Slc5a5 ^a^
Thrombin receptor signaling pathway	1.3 × 10^−5^	1.1 × 10^−3^	F2rl2	
Positive regulation of exocytosis	1.7 × 10^−5^	1.3 × 10^−3^	Cacna1h, F2rl2	Zp3
Response to potassium ion	3.8 × 10^−5^	2.3 × 10^−3^	Cacna1h	
Membrane depolarization	6.6 × 10^−5^	3.3 × 10^−3^	Cacna1h, Cacng1	Hrh2
Diseases (in biomarkers)				
Epilepsy, absence	2.0 × 10^−6^	1.5 × 10^−3^	Cacna1h, Cacng1	
Epilepsy, generalized	1.3 × 10^−5^	4.7 × 10^−3^	Cacna1h, Cacng1	
Seizures	4.0 × 10^−5^	7.7 × 10^−3^	Cacna1h, Cacng1	
Autistic disorder	4.9 × 10^−5^	7.7 × 10^−3^	Cacna1h, F2rl2	Hrh2, Htr2c, Tacr3
Child development disorders, pervasive	5.2 × 10^−5^	7.7 × 10^−3^	Cacna1h, F2rl2	Hrh2, Htr2c, Tacr3
Mental disorders diagnosed in childhood	1.5 × 10^−4^	1.8 × 10^−2^	Cacna1h, F2rl2	Hrh2, Htr2c, Tacr3
Epilepsy	1.2 × 10^−3^	6.9 × 10^−2^	Cacna1h, Cacng1	Hrh2, Htr2c, Tacr3, Ihh ^c^
Mitochondrial complex I deficiency	2.0 × 10^−3^	7.8 × 10^−2^	Ndufaf2	
Delirium	3.0 × 10^−3^	8.0 × 10^−2^	F2rl2	
Neurologic manifestations	4.8 × 10^−3^	8.4 × 10^−2^	Cacna1h, Cacng1, F2rl2	Hrh2, Htr2c, Dcdc2 ^a^, Magel2 ^b^, Pitx3 ^b^, Acta1 ^c^, Actc1 ^a^

^a^ DE gene in CvA; ^b^ DE gene in CvI; ^c^ DE gene in CvN. Enrichment analyses of the Compare Experiments Workflow of MetaCore. Significant categories are listed. This tool can be used for comparing experimental data by analyzing their intersections in terms of their mappings onto MetaCore’s various ontologies. DE: differentially expressed; CvN: control versus NIC; CvA: control versus ACE; CvI: control versus IMI; FDR: false discovery rate; GO: gene ontology.

## Supplementary Materials

The following are available online at http://www.mdpi.com/1660-4601/13/10/987/s1, Figure S1: Percentages of cell types in cerebellar neuron-enriched cultures; Table S1: Oligonucleotide primers used for qRT-PCR in the 5’-3’ direction; Table S2: Standard deviations and standard errors of DE genes for CvN, CvA, and CvI; Table S3: Detailed classification of DE genes for CvN, CvA, and CvI by PANTHER.

## Abbreviations

NIC	nicotine
IMI	imidacloprid
ACE	acetamiprid
nAChR	nicotinic acetylcholine receptor
ACh	acetylcholine
BBB	blood brain barrier
GFAP	glial fibrillary acidic protein
qRT-PCR	quantitative real-time PCR
DIV	days in vitro
DE	differentially expressed
FC	fold change
GO	gene ontology
FDR	false discovery rate
CvN	control versus NIC
CvA	control versus ACE
CvI	control versus IMI
GPCR	G-protein coupled receptor
