# Supplementary material for: Neonicotinoid Insecticides Alter the Gene Expression Profile of Neuron-Enriched Cultures from Neonatal Rat Cerebellum
Source: Int J Environ Res Public Health. 2016 Oct 4;13(10):987. doi: 10.3390/ijerph13100987 (PMC5086726; doi:10.3390/ijerph13100987)
Supplement: Supplementary file 1 [file ijerph-13-00987-s001.pdf]

Supplementary Materials: Neonicotinoid Insecticides Alter the Gene Expression Profile of Neuron-Enriched Cultures from Neonatal Rat Cerebellum

Junko Kimura-Kuroda, Yasumasa Nishito, Hiroko Yanagisawa, Yoichiro Kuroda, Yukari Komuta, Hitoshi Kawano and Masaharu Hayashi

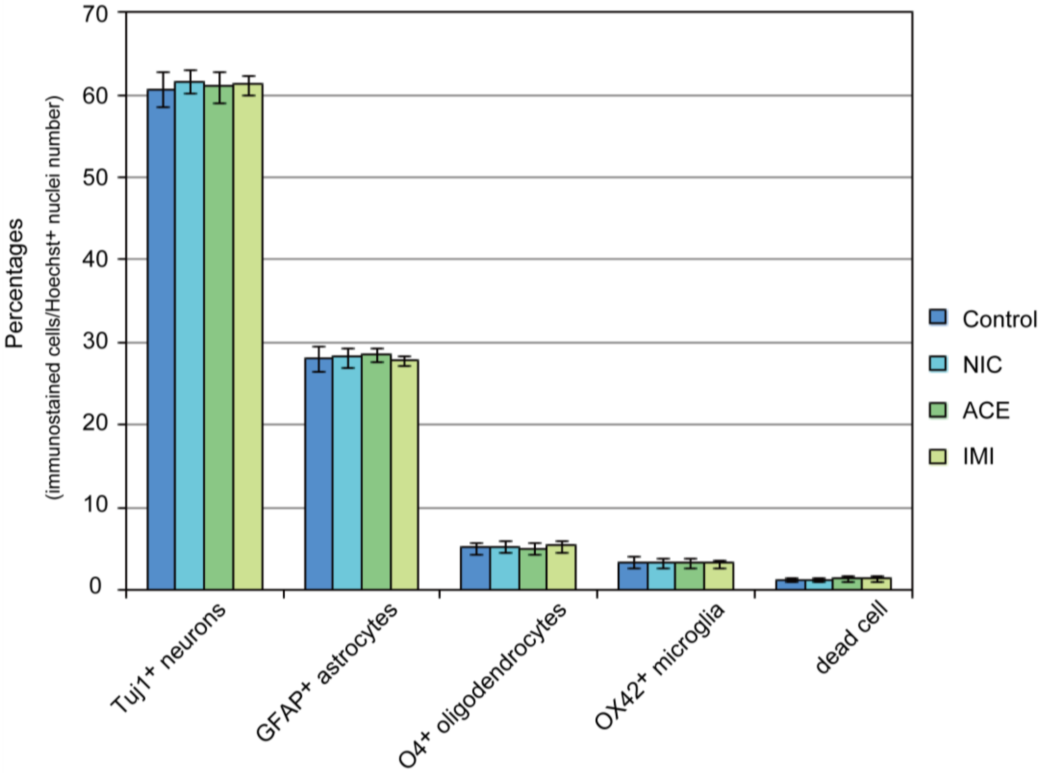

Figure S1. Percentages of cell types in cerebellar neuron-enriched cultures. At 16 DIV, the cultured cerebellar cells were fixed and stained with specific neural antibodies; mouse monoclonal anti-Tuj1 as a neuronal marker, rabbit anti-GFAP as an astrocyte marker, mouse anti-oligodendrocyte marker O4, mouse anti-CD11b OX42 IgM as a microglial marker, and Hoechst 33342 as a nucleus. The percentages were calculated from the number of immnostained cells per Hoechst$^+$-nuclei number (n = approximately 1000 per treatment group per experiment) of three to four experiments, using MetaMorph imaging software. Error bars show standard deviations. No significant differences of neural cell percentages were observed between control and nicotine (NIC)-, acetamiprid (ACE)-, and imidacloprid (IMI)-treatments.

Table S1. Oligonucleotide primers used for qRT-PCR in the 5′–3′ direction.

Gene Name	Sense	Antisense	Product (Base Pair)	Accession Number
B3gnt9	GGTTTGACCAGCACAGAAGC	GGGAAGCCAATGTAGAGAAGG	189	XM_226431
Cadm3	CCCTCAAGATGACCCAAGAG	CAGCAGGAAGACGATGAAAG	209	NM_001047103
Lyn	TTGAACCGAAGTCACCGTGG	ATTCAGGAACTGGCCTTTGC	201	NM_030857
Magel2	CCAAAGGCAACAGACAATGG	GACACCAAACAGGGAACCAC	214	XM_001054803
Rbfox2	GTAAATCCACGCCCAAGC	GCCAAACTGCCCAAACATC	89	NM_001079895
Actb	CCTCTATGCCAACACAGTGC	GTACTCCTGCTTGCTGATCC	211	NM_031144
Gapdh	GACATGCCGCCTGGAGAAAC	AGCCCAGGATGCCCTTTAGT	92	NM_017008

For Atp5f1, Bche, Cacna1h, Cacng1, Dcdc2, F2rl2, Gpr83, Htr2c, Mb, Mcmdc2, Myog, Mypn, Pcdhgb7, Tada2b, Tnni2, Txk, Unc45b, Chrna3, Chrna4, and Chrna7, ready-made primers were purchased from TAKARA Perfect Real Time PCR System (TAKARA, Tokyo, Japan).

Table S2. Standard deviations and standard errors of 34 DE genes for CvN, CvA, and CvI.

Gene Symbol	*n*	log2 FC	SD	SE
**34 DE genes for CvN**				
Acta1	6	−1.255	1.330	0.543
Actrt2	6	−0.933	1.446	0.590
Ankrd60	6	−0.969	1.057	0.431
Atf7	6	0.664	0.882	0.360
Cacna1h	6	0.737	0.353	0.144
Cacng1	6	−1.080	0.980	0.400
Cadm3	6	0.611	0.759	0.310
Car3	6	−0.753	0.689	0.281
Cd86	6	0.602	0.525	0.214
Celf6	6	0.724	0.583	0.238
Cramp1l	6	1.218	1.268	0.518
Cyp17a1	6	−0.590	0.632	0.258
F2rl2	6	1.023	1.279	0.522
Gpr83	6	0.752	0.455	0.186
Gramd4	6	0.708	1.101	0.450
Ihh	6	0.911	1.012	0.413
Kcnq5	6	1.864	1.782	0.728
Klhl18	6	0.624	0.342	0.139
Lmod3	6	−2.013	1.439	0.587
LOC317356	6	1.065	1.323	0.540
Mcmdc2	6	0.733	0.481	0.196
Ms4a12	6	−0.589	0.393	0.161
Mypn	6	−0.661	0.453	0.185
Ndufaf2	6	−0.785	0.615	0.251
Nhlh2	6	−0.678	0.459	0.187
Nt5dc1	6	0.661	0.803	0.328
Olr1051	6	−1.275	1.409	0.575
Pcdhgb7	6	0.951	1.139	0.465
Phldb3	6	−0.845	1.265	0.517
Sdr42e2	6	−0.783	0.637	0.260
Tada2b *	5 *	0.661	0.453	0.203
Unc45b	6	−0.747	0.849	0.347
Vsig2	6	0.635	0.836	0.341
Zp3	6	−0.822	0.944	0.385
**48 DE genes for CvA**				
Actc1	6	−1.139	1.427	0.583
Actrt2	6	−1.079	1.193	0.487
Ankrd60	6	−0.874	1.234	0.504
Asap2	6	0.636	0.403	0.164
B3gnt9	6	1.805	1.934	0.789
Cacna1h	6	0.706	0.378	0.154
Cacng1	6	−0.991	1.127	0.460
Cramp1l	6	0.733	1.003	0.409
Dcdc2	6	−0.760	1.093	0.446
Des	6	−1.636	2.106	0.860
Dmbt1	6	−0.888	1.248	0.509
Dupd1	6	1.838	1.871	0.764
F2rl2	6	0.892	0.900	0.368
Fyb	6	0.587	0.615	0.251
Hrh2	6	1.292	0.965	0.394
Hsd3b1	6	−0.907	0.870	0.355
Hspb7	6	−1.067	1.306	0.533
Iqcf1	6	−0.601	0.812	0.332
Lmod2	6	−1.633	1.828	0.746

Table S2. *Cont.*

Gene Symbol	*n*	log2 FC	SD	SE
**48 DE genes for CvA**				
Lmod3	6	−1.747	1.460	0.596
LOC100363332	6	0.714	0.710	0.290
LOC499235	6	0.643	0.958	0.391
LOC679149	6	0.748	0.571	0.233
LOC684762	6	−1.492	2.142	0.874
Lyn	6	1.116	1.561	0.637
Mb	6	−1.103	0.945	0.386
Mcmdc2	6	1.168	1.851	0.756
Mroh1	6	0.601	0.509	0.208
Myog	6	−0.740	0.779	0.318
Nags	6	−1.046	1.296	0.529
Napb	6	0.660	0.610	0.249
Ndufaf2	6	−0.691	0.543	0.222
Ntn1	6	0.692	0.384	0.157
Olr1714	6	−0.800	0.750	0.306
Olr825	6	−0.602	0.395	0.161
Olr94	6	−0.705	1.129	0.461
Phf21b	6	0.718	0.803	0.328
Plac8	6	0.649	0.524	0.214
Rasl10b	6	0.679	0.468	0.191
Sdr42e2	6	−0.856	0.826	0.337
Slc45a3	6	1.109	1.591	0.650
Slc5a5	6	−0.678	0.543	0.222
Slco1b2	6	−1.415	1.668	0.681
Tada2b	6	1.193	1.171	0.478
Tnni2	6	−0.943	1.462	0.597
Unc45b	6	−0.839	0.939	0.383
Vom1r73	6	−0.592	0.620	0.253
Zfp498	6	0.662	0.734	0.300
**67 DE genes for CvI**				
Amer3	6	−0.717	0.735	0.300
Ampd1	6	−0.827	0.826	0.337
Bmp8b	6	−0.668	0.736	0.301
C2cd4c	6	−0.651	1.088	0.444
Cacna1h	6	0.640	0.468	0.191
Cacng1	6	−1.043	0.874	0.357
Ccdc81	6	0.616	0.677	0.276
Cdc6	6	0.676	1.000	0.408
Celf6	6	0.629	0.508	0.207
Cramp1l	6	0.691	0.901	0.368
Crb3	6	0.888	0.610	0.249
Cxcl17	6	0.695	0.739	0.302
Cyp17a1	6	−0.730	0.953	0.389
Cyp4a1	6	−0.654	0.980	0.400
Efna3	6	0.695	0.932	0.380
Egr3	6	−0.772	0.751	0.307
F2rl2 *	5 *	0.864	0.928	0.415
Galnt7	6	0.659	0.896	0.366
Gapt	6	−0.712	0.775	0.317
Grb7	6	0.768	0.546	0.223
Hao1	6	−0.798	0.926	0.378
Hfe2	6	−1.674	1.658	0.677
Hlcs	6	−0.586	0.573	0.234

Table S2. *Cont.*

Gene Symbol	*n*	log2 FC	SD	SE
**67 DE genes for CvI**				
Hspb7	6	−1.303	0.928	0.379
Htr2c	6	1.607	1.405	0.574
Iqsec3	6	0.619	0.253	0.103
Itpripl1	6	−0.792	1.495	0.610
Klk1c7	6	−1.221	1.553	0.634
Krt4	6	−0.833	1.029	0.420
Krt5	6	−0.662	0.888	0.362
Lbx1	6	1.097	0.700	0.286
Lmod3	6	−1.647	1.365	0.557
LOC100364862	6	−0.885	0.821	0.335
LOC100912563	6	−1.193	1.340	0.547
LOC294497	6	−0.614	0.799	0.326
LOC685406	6	−0.763	0.843	0.344
Lyn	6	0.730	0.465	0.190
Magel2	6	0.629	0.286	0.117
Mb	6	−0.822	0.709	0.290
Mrgprb4	6	−0.646	0.667	0.272
Mybpc2	6	0.636	0.690	0.282
Mypn	6	−0.711	0.604	0.247
Ndufaf2	6	−0.801	0.699	0.285
Neurog3	6	−0.753	0.890	0.364
Oasl	6	0.768	0.731	0.298
Pcdhgb7	6	0.614	0.780	0.318
Pitx3	6	−0.764	1.033	0.422
Plekha2	6	0.746	1.172	0.479
Prr16	6	−0.889	0.852	0.348
Rbfox2	6	0.668	0.926	0.378
RGD1561114	6	−0.621	1.142	0.466
RGD1564095	6	−0.882	0.896	0.366
RGD1564571	6	−0.610	0.665	0.271
RGD1566248	6	−1.170	1.397	0.570
S100pbp	6	0.613	0.943	0.385
Scnn1b	6	−1.022	1.033	0.422
Sdr42e2	6	−0.809	0.761	0.311
Slc2a3	6	0.740	0.530	0.216
Slc38a8	6	−0.769	1.007	0.411
Syne4	6	1.013	1.484	0.606
Tacr3	6	0.862	1.015	0.414
Tada2b	6	1.049	0.846	0.345
Tmem52b	6	−0.700	1.124	0.459
Tnni2	6	−0.847	0.902	0.368
Txk	6	0.712	0.906	0.370
Unc45b	6	−0.653	0.579	0.237
Zp3	6	−1.078	1.171	0.478

CvN: control versus nicotine; CvA: control versus acetamiprid; CvI: control versus imidacloprid; SD: standard deviation; SE; standard error. * For Tada2b in CvN, and F2rl2 in CvI, one of six microarrays was compromised and excluded.

Table S3. Detailed classification of DE genes for CvN, CvA, and CvI by PANTHER.

Category of GO Biological Processes	Number (%)	Gene Name
**CvN**		
biological adhesion (GO:0022610)	3 (5.2)	Cadm3, Pcdhgb7, Mypn
biological regulation (GO:0065007)	5 (8.6)	Tada2b, Kcnq5, Cacna1h, Atf7, Nhlh2
cellular component organization or biogenesis (GO:0071840)	3 (5.2)	Actrt2, Lmod3, Acta1
cellular process (GO:0009987)	17 (29.3)	Ms4a12, Tada2b,Kcnq5, Gpr83, Actrt2, Lmod3, Cadm3, Acta1, Mcmdc2, Cacna1h, Atf7, Nt5dc1, Pcdhgb7, Vsig2, F2rl2, Mypn, Phldb3
developmental process (GO:0032502)	8 (13.8)	Actrt2, Lmod3, Celf6, Acta1, Cd86, Pcdhgb7, Mypn, Phldb3
immune system process (GO:0002376)	2 (3.4)	Gpr83, Phldb3
localization (GO:0051179)	4 (6.9)	Kcnq5, Actrt2, Acta1, Cacna1h
metabolic process (GO:0008152)	8 (13.8)	Tada2b, Mcmdc2, Atf7, Nt5dc1, Nhlh2, Cyp17a1, Unc45b, Mypn
multicellular organismal process (GO:0032501)	4 (6.9)	Gpr83, Lmod3, Celf6, Mypn
response to stimulus (GO:0050896)	4 (6.9)	Ms4a12, Gpr83, Unc45b, Phldb3
10 categories for CvN	Total 58 (100)	
**CvA**		
apoptotic process (GO:0006915)	1 (1.1)	Lyn
biological adhesion (GO:0022610)	3 (3.2)	Ntn1, Rasl10b, Lyn
biological regulation (GO:0065007)	7 (7.5)	Olr1714, Tada2b, Asap2, Myog, Lyn, Cacna1h, Phf21b
cellular component organization or biogenesis (GO:0071840)	8 (8.6)	Myog, Actrt2, Lmod3, Napb, Lyn, Lmod2, Des, Actc1
cellular process (GO:0009987)	20 (21.5)	B3gnt9, Olr1714, Tada2b, Ntn1, Asap2, Rasl10b, Fyb, Myog, Actrt2, Lmod3, Napb, Lyn, Mcmdc2, Cacna1h, Dmbt1,Dcdc2, F2rl2, Lmod2, Des, Actc1
developmental process (GO:0032502)	10 (10.8)	B3gnt9, Ntn1, Myog, Actrt2, Lmod3, Lyn, Tnni2, Lmod2, Des, Actc1
immune system process (GO:0002376)	3 (3.2)	Lyn, Dmbt1, Dcdc2
localization (GO:0051179)	11 (11.8)	Rasl10b, Actrt2, Slco1b2, Napb, Lyn, Cacna1h, Dmbt1, Mb, Slc5a5, Slc45a3, Actc1
locomotion (GO:0040011)	1 (1.1)	Lyn
metabolic process (GO:0008152)	15 (16.1)	B3gnt9, Tada2b, Asap2, Rasl10b, LOC679149, Myog, Nags, Hsd3b1, Lyn, Mcmdc2, Dmbt1, Slc5a5, Slc45a3, Phf21b, Unc45b
multicellular organismal process (GO:0032501)	10 (10.8)	B3gnt9, Olr1714, Ntn1, Rasl10b, Myog, Lmod3, Lyn, Tnni2, Mb, Lmod2
reproduction (GO:0000003)	1 (1.1)	B3gnt9
response to stimulus (GO:0050896)	3 (3.2)	Olr1714, Lyn, Unc45b
13 categories for CvA	Total 93 (100)	
**CvI**		
apoptotic process (GO:0006915)	3 (2.4)	Magel2, Bmp8b, Lyn
biological adhesion (GO:0022610)	4 (3.2)	RGD1564571, Lyn, Pcdhgb7, Mypn
biological regulation (GO:0065007)	15 (11.9)	Lbx1, Efna3, Tada2b, Txk, Hfe2, Neurog3, Scnn1b, Bmp8b, RGD1561114, Tacr3, Lyn, Pitx3, Cacna1h, RGD1566248, Crb3
cellular component organization or biogenesis (GO:0071840)	6 (4.8)	Krt4, Efna3, Iqsec3, Krt5, Lmod3, Lyn
cellular process (GO:0009987)	24 (19.0)	Krt4, RGD1564571, Efna3, Tada2b, Txk, Iqsec3, Krt5, Hfe2, Lmod3, Magel2, Bmp8b, RGD1561114, Tacr3, Lyn, Grb7, Cacna1h, Slc38a8, Pcdhgb7, F2rl2, Mrgprb4, Mypn, Crb3, Htr2c, Cdc6
developmental process (GO:0032502)	16 (12.7)	Krt4, Lbx1, Efna3, Txk, Krt5, Lmod3, Neurog3, Magel2, Bmp8b, Celf6, Lyn, Pitx3, Tnni2, Pcdhgb7, Mypn, Crb3
growth (GO:0040007)	1 (0.8)	Txk
immune system process (GO:0002376)	5 (4.0)	RGD1564571, Oasl,Txk, Lyn, Crb3
localization (GO:0051179)	7 (5.6)	RGD1564571, Scnn1b, Lyn, Cacna1h, Mb, Slc38a8, Crb3
locomotion (GO:0040011)	2 (1.6)	Efna3, Lyn
metabolic process (GO:0008152)	22 (17.5)	Lbx1, Hao1, Oasl, Tada2b, Txk, Hlcs, Hfe2, Neurog3, Bmp8b, RGD1564095, RGD1561114, Tacr3, Lyn, Pitx3, RGD1566248,, Galnt7, Ampd1, Cyp17a1, Unc45b, Mypn, Crb3, Cdc6
multicellular organismal process (GO:0032501)	11 (8.7)	Efna3, Txk, Lmod3, Scnn1b, Celf6, Lyn, Pitx3, Tnni2, Mb, Mypn, Htr2c
reproduction (GO:0000003)	1 (0.8)	Magel2

Table S3. *Cont*.

Category of GO Biological Processes	Number (%)	Gene Name
**CvI**		
response to stimulus (GO:0050896)	9 (7.1)	RGD1564571, Efna3, Oasl, Txk, Hfe2, Bmp8b, Tacr3, Lyn, Unc45b
14 categories for CvI	Total 126 (100)	

DE: differentially expressed; CvN: control versus nicotine; CvA: control versus acetamiprid; CvI: control versus imidacloprid; GO: gene ontology.

© 2016 by the authors. Submitted for possible open access publication under the terms and conditions of the Creative Commons Attribution (CC-BY) license (http://creativecommons.org/licenses/by/4.0/).
